# EEG hyperscanning in intellectual disability: a scoping review with implications for cognitive stimulation therapy

**DOI:** 10.3389/fnrgo.2026.1757738

**Published:** 2026-04-13

**Authors:** Pavithra Pavithra, James K. Bradshaw Bernacchi, Salma Ahmed, Garret McDermott, Mary McCarron, Philip McCallion, Eimear McGlinchey, Alejandro Lopez Valdes

**Affiliations:** 1 Trinity Centre for Ageing and Intellectual Disability, School of Nursing and Midwifery, Trinity College Dublin, The University of Dublin, Dublin, Ireland; 2 Department of Electronic and Electrical Engineering, School of Engineering, Trinity College Dublin, The University of Dublin, Dublin, Ireland; 3 Trinity Centre for Biomedical Engineering, Trinity College Dublin, The University of Dublin, Dublin, Ireland; 4 Trinity College Institute of Neuroscience, Trinity College Dublin, The University of Dublin, Dublin, Ireland; 5 Department of Psychology, Tallaght University Hospital, Dublin, Ireland; 6 Temple School of Social Work, College of Public Health, Temple University, Philadelphia, PA, United States; 7 Global Brain Health Institute, Trinity College Dublin, The University of Dublin, Dublin, Ireland

**Keywords:** Alzheimer's disease, cognitive stimulation therapy, Down syndrome, EEG, hyperscanning, intellectual disability

## Abstract

Electroencephalography (EEG) hyperscanning has emerged as a valuable method for examining social dynamics during group-based activities and may serve as a promising outcome measure in group interventions. Cognitive stimulation therapy (CST) is one such interventions shown to improve cognition and quality of life in people with dementia and has recently been adapted for individuals with intellectual disability (ID). However, the potential for obtaining objective neural markers of CST benefit via EEG and hyperscanning is yet to be explored. This scoping review aims to identify existing evidence and gaps related to the use of EEG within CST research for adults with ID by examining three relevant areas: (1) the use of individual EEG and hyperscanning to evaluate cognitive and social outcomes in CST; (2) the evidence base for individual and group-based CST in people with ID; and (3) the use of EEG to evaluate cognitive and social outcomes for people with ID. Following the PRISMA-ScR guidelines, studies were searched in CINAHL, MEDLINE, PsychInfo, and EMBASE. Our search focused on adult participants with ID and studies that used EEG for the purpose of evaluating cognitive or social outcomes. Currently, there are no studies that use EEG to evaluate CST in adults with ID. Following screening and eligibility assessment, no studies met the inclusion criteria for EEG and CST. Five studies were included for CST and ID, and 14 articles met criteria for EEG and ID. In total, 19 articles were included in the final review. The evidence base suggests that EEG has been successfully used to investigate neural mechanism in ID and Down Syndrome related Alzheimer's disease. Existing CST research in ID remains largely feasibility-focused but some preliminary findings show cognitive benefits, enhanced enjoyment, and social connectedness. Our review shows that there is a large gap when it comes to any objective metrics for CST in general. Given that there is evidence of EEG studies including populations with ID, we propose that this gap can be filled by EEG hyperscanning which offers a non-invasive, objective approach to evaluate cognitive and social outcomes in people with ID in future CST research.

## 1 Introduction

Intellectual disability (ID) is marked by significant limitations in intellectual functioning and adaptive functioning with onset during the developmental period, affecting the conceptual, social, and practical skills required for everyday life (World Health Organization, 2019). It is estimated to affect approximately 1.7% of the North American population and about 3% of the global population, with the highest regional prevalence in South Asia (~5.6%) (Olusanya et al., 2022). With improved healthcare and community-based support, people with ID now experience greater lifespan and quality of life, alongside new challenges such as increased comorbidity burden (Kumar, 2024; McCarron et al., 2015; Ryan et al., 2025). Dementia is one such challenge, with prevalence in people with ID estimated to be five times higher than in the general population (Strydom et al., 2013). For people with Down syndrome (DS), the lifetime risk of developing Alzheimer's Disease (AD) is over 95% (McCarron et al., 2017), due to the triplication of the amyloid precursor protein (APP) gene, leading to overproduction of amyloid- ß plaques and universal neuropathology of AD by age 40 (Fortea et al., 2021).

Lower cognitive reserve puts people with ID at a higher risk for dementia as they age (Strydom et al., 2009), with lifelong disparities faced by compounding this further (Fortea et al., 2024; McMahon et al., 2024). Despite this elevated risk of dementia, research on post-diagnostic dementia support for people with ID is limited (Dennehy et al., 2023), as are cognitive and social interventions to reduce lifestyle risk factors. Cognitive Stimulation Therapy (CST) (Spector et al., 2003) is an evidence-based dementia intervention shown to enhance both cognition and quality of life in the general population (Saragih et al., 2022) and has recently been adapted for people with ID (Ali et al., 2025; Hardiman et al., 2025). CST involves themed activities grounded in 18 key principles which are person-centered, involvement, choice, respect, inclusion, fun, maximizing potential, mental stimulation, opinions rather than facts, using the senses, using reminiscence, triggers for recall, stimulating language, stimulating executive functioning, orientation, continuity and consistency, implicit learning, and building/strengthening relationships. This structure has enabled it to improve cognition, confidence, enjoyment, mood, and interaction amongst participants, as reported in the literature (Gibbor et al., 2021). Originally, CST was developed as a group intervention with 14 sessions conducted for 45 min over the period of 7-weeks in a twice a week session style. The 45 min is divided into 10 min of introduction, 25 min of main activity, and 10 min of summarizing the session. The introduction involves singing the group song, playing a short physical game such as bean bag toss, discussing the date, time, month, and venue and discussing easy-read news. This segment mainly promotes continuity and orientation amongst other key principles mentioned. The 25-min main activity revolves around the theme of the session. There are 14 themes which are followed according to the session number. The 14 themes are physical games, sound, childhood, food, current affairs, faces and scenes, word association, being creative, categorizing objects, orientation, number games, word games, and team quiz which can involve repeat of a favorite session along with a party to celebrate the end of intervention. Complementary variants of CST have also been developed. A 24-week maintenance programme has been implemented to extend the benefits from the main interventions (Aguirre et al., 2010). Additionally, CST has been adapted as a shorter 25-week individual programme that can be delivered by a caregiver (Orrell et al., 2017).

CST is an emerging topic in ID literature and as an emerging area of research, published studies to date have primarily focused on feasibility. While some studies have used validated scales and qualitative interviews to assess outcomes related to cognition, quality of life, and adaptive behavior, there remains a notable gap in incorporating objective outcome measures. Assessing cognition and social interactions in this population is particularly challenging due to pre-existing ID coupled with the progression of dementia. Studies have found that traditional measurement scales are also subject to limitations, including floor effects (Orsini et al., 2015) and ceiling effects (Aguayo et al., 2019), whereby scores cluster at the lower or upper extremes of the scale. Objective measures, defined as the quantification of variables using instrument that produce consistent and reproducible values independent of subjective interpretation, may help to address these limitations as well as issues such as acquiescence bias and difficulties completing standardized assessments. In this context, the inclusion of an objective neurophysiological measure may complement traditional assessments and strengthen the robustness of outcome evaluation.

Electroencephalography (EEG) is an electrophysiological method for measuring brain electrical activity. Traditionally, it has been widely utilized because it is affordable, non-invasive, and offers high temporal resolution for tracking neural dynamics. Accordingly, EEG is used to characterize and diagnose brain disorders (e.g., epilepsy, ADHD) (Benbadis et al., 2020), to monitor brain activity (Musaeus et al., 2022), and to investigate the neural processes of the brain (Michel and Koenig, 2018). Moreover, it is extensively employed as an outcome measure in studies on cognitive training (Fabio et al., 2016; Gandelman-Marton et al., 2017; Santarnecchi et al., 2017) and other psychological interventions (Zou, 2022). Such studies are commonly performed in the general population, with very few conducted in populations with ID. However, some research has been conducted in relation to individuals with ID, highlighting EEG as a promising objective method for future trials investigating psychosocial interventions. EEG hyperscanning (from now on referred simply as hyperscanning) has emerged as a method to simultaneously record brain activity in two or more individuals, allowing to investigate the underpinnings of social dynamics in both laboratory and naturalistic settings (Czeszumski et al., 2020; Liu et al., 2018). Hyperscanning may provide additional insights into cognitive and social processes relevant to CST and could contribute to the development of more objective outcome measures for psychosocial interventions.

Despite EEG being a well-established method to investigate social and cognitive outcomes, limited work has been published in people with ID, especially on its use in CST. This scoping review aims to map the existing evidence by addressing the following research questions (RQ):

- RQ1- How has EEG (including hyperscanning) been used to evaluate cognitive and social outcomes in CST?
- RQ2- What is the evidence base for individual and group CST for people with ID?
- RQ3- How has EEG been used to evaluate cognitive and social outcomes for people with ID?

This scoping review aims primarily to map existing evidence on CST and EEG in people with ID. In addition, it examines how EEG, including hyperscanning, has been reported in related populations to inform preliminary conceptual framework for future research. The review highlights gaps in the evidence base and considerations for future studies.

## 2 Materials and methods

### 2.1 Design

This scoping review was conducted in accordance with the Preferred Reporting Items for Systematic Reviews and Meta-Analyses extension for Scoping Reviews (PRISMA-ScR) guidelines (Tricco et al., 2018) and followed the Joanna Briggs Institute (JBI) methodological framework for scoping reviews (Aromataris et al., 2024).

### 2.2 Search strategy

A comprehensive search was conducted in August 2025 by one reviewer (PP) across CINAHL, MEDLINE, PsycINFO, and Embase. The search strategy operationalised the research aim by separating it into three complementary query streams aligned with the research questions: (RQ1) EEG AND CST, (RQ2) CST AND ID, and (RQ3) EEG AND ID. These streams were chosen to (i) capture any EEG work conducted in CST contexts, (ii) characterize CST evidence in people with ID, and (iii) identify EEG evidence in people with ID that could inform feasibility and methodological considerations for future CST hyperscanning work.

Within each stream, synonyms were combined using OR and concepts were combined using AND. Searches were limited to Title/Abstract fields and were adapted to each database's syntax (including wildcard use). Table 1 reports the search term blocks and synonyms used for the database searches (i.e., the concept groups combined to form each stream query). Two additional records (including one from gray literature) were identified in September 2025 through citation searching. See Supplementary Table S1 for the extended search strategy.

**Table 1:** Search concept blocks and synonyms (Title/Abstract).

Concept block	Title/Abstract search terms
EEG	(“Electroencephalography” OR “EEG” OR “Electroencephalogram”)
Cognitive stimulation therapy	(“Cognitive stimulation therapy” OR “CST”)
Intellectual disability	(“Intellectual disability” OR “person with intellectual disability” OR “learning disability” OR “Down syndrome” OR “Intellectual development disorder” OR “Intellectual impairment”)

Within-block terms were combined with OR and blocks were combined with AND to build the query streams.

### 2.3 Selection process

**Figure 1:**
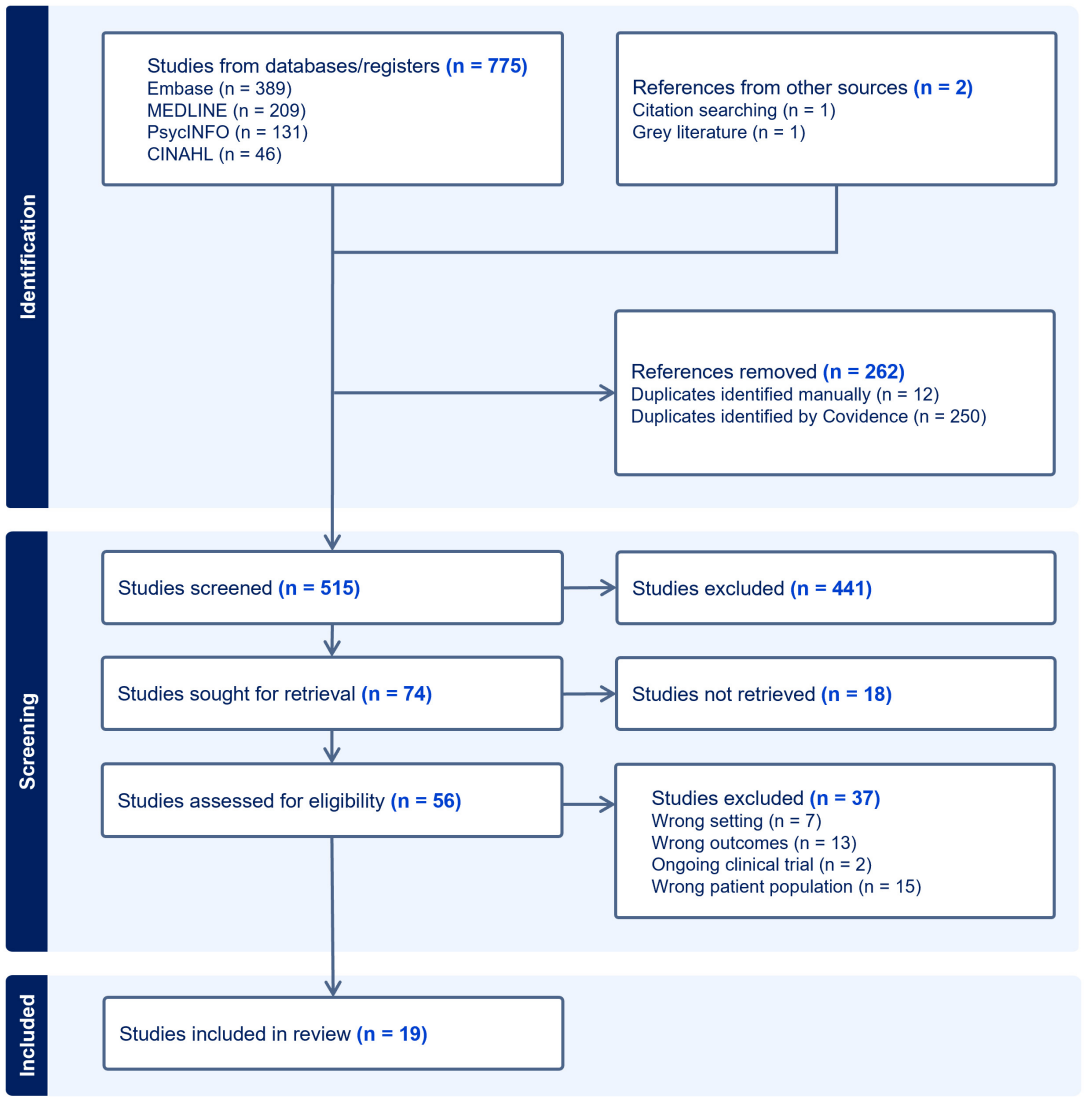
PRISMA flow diagram (combined across the three research questions: EEG-CST, CST-ID, and EEG-ID).

Records were retrieved and screened from the three query streams: (RQ1) EEG AND CST, (RQ2) CST AND ID, and (RQ3) EEG AND ID with specific inclusion and exclusion criteria for each stream. All retrieved articles were imported into Covidence, and duplicates were removed. Two reviewers (PP and JB) independently screened titles and abstracts, and then full texts against the eligibility criteria. Disagreements were resolved through discussion. Quality assurance and bias reduction were maintained by involving two independent reviewers PP and JB at each stage, with regular meetings to resolve any discrepancies. Figure 1 shows the PRISMA flow diagram for the three combined research questions. PRISMA diagrams specific to each research question are provided in the Supplementary material (see Supplementary Figures S1–S3).

### 2.4 Eligibility criteria

This scoping review included articles relevant to each of the three research questions; accordingly, some inclusion/exclusion criteria are specific to each RQ. Global inclusion criteria were: full-length articles; studies published between 2000 and 2025; English language; and human participants aged 16 years or older. Global exclusion criteria were: studies involving only animal models or participants under 16 years old; records with only abstracts, theses dissertations, protocol papers, or any item without full text available.

Specific inclusion criteria for RQ1 included the use of EEG as the neuroimaging method and CST interventions. RQ2 included participants with ID and CST interventions. RQ3 included the use of EEG as a neuroimaging method, and participants with ID (including those with dementia). Participants with learning disorders (e.g., dyslexia) were not included unless it was explicitly stated that they also had ID. Articles employing only non-EEG neuroimaging methods and studies with only neurotypical participants were excluded. For RQ1 and RQ3, inclusion required outcomes related to cognition, quality of life, or social facilitation; therefore, studies with different outcomes (e.g., those focusing solely on epilepsy or sleep) were excluded. One review article related to EEG in ID was included as it provided insightful interpretations; no references were double counted to keep consistency with the retrieved results (Victorino et al., 2023).

### 2.5 Data extraction

The extraction template contained:

- General information: study ID, title, author(s), year of publication, and country of origin.
- General methods: aim of study and study design.
- Population: age, gender, sample size, population group, and inclusion and exclusion criteria.
- Outcome measures: assessments related to cognition, quality of life and other types of tests. For RQ3, EEG metrics used to analyse the data were also included in this subsection.
- Results: key findings that relate to the scoping review question/s (e.g., feasibility findings and outcome results).

For RQ3, the extraction template also contained a subsection in methods related to EEG specifications (electrode type, EEG modality, and spatial configuration). Additionally, details of the EEG recording protocol were also included in this subsection, including the tasks performed during acquisition (e.g., resting state). See Supplementary Tables S2, S3 for data extraction templates.

**Figure 2:**
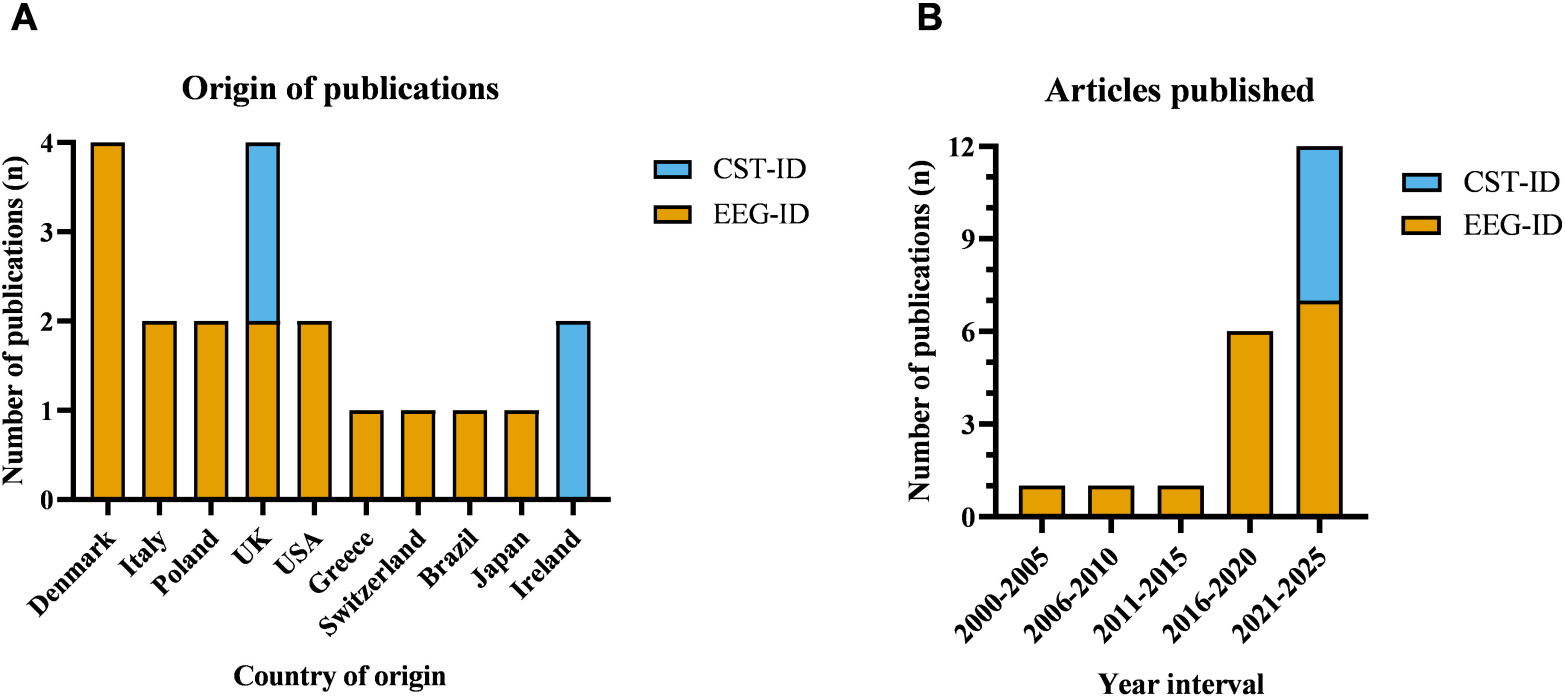
Geographic and temporal distribution of included studies. **(A)** Country of origin for publications on EEG in intellectual disability (EEG-ID, orange) and Cognitive Stimulation Therapy in intellectual disability (CST-ID, blue). Most records originated from the UK and Denmark, with additional contributions from Italy, Poland, the USA, Greece, Switzerland, Brazil, Japan, and Ireland. **(B)** Publications by 5-year interval for EEG-ID (orange) and CST-ID (blue). Output rose sharply in 2021–2025 (EEG-ID *n* = 7; CST-ID *n* = 5), following sparse publications in earlier periods.

PP and JB completed data extraction, with cross validation of 20% of the extracted data to ensure accuracy and consistency. Extracted data was exported as a CSV into Microsoft Excel (Version 2502 Build 16.0.18526.20546) (Microsoft Corporation, Redmond, Washington, USA) and used to generate figures and tables. Figure 2 below shows origin and year of publication across included studies.

## 3 Results

**Figure 3:**
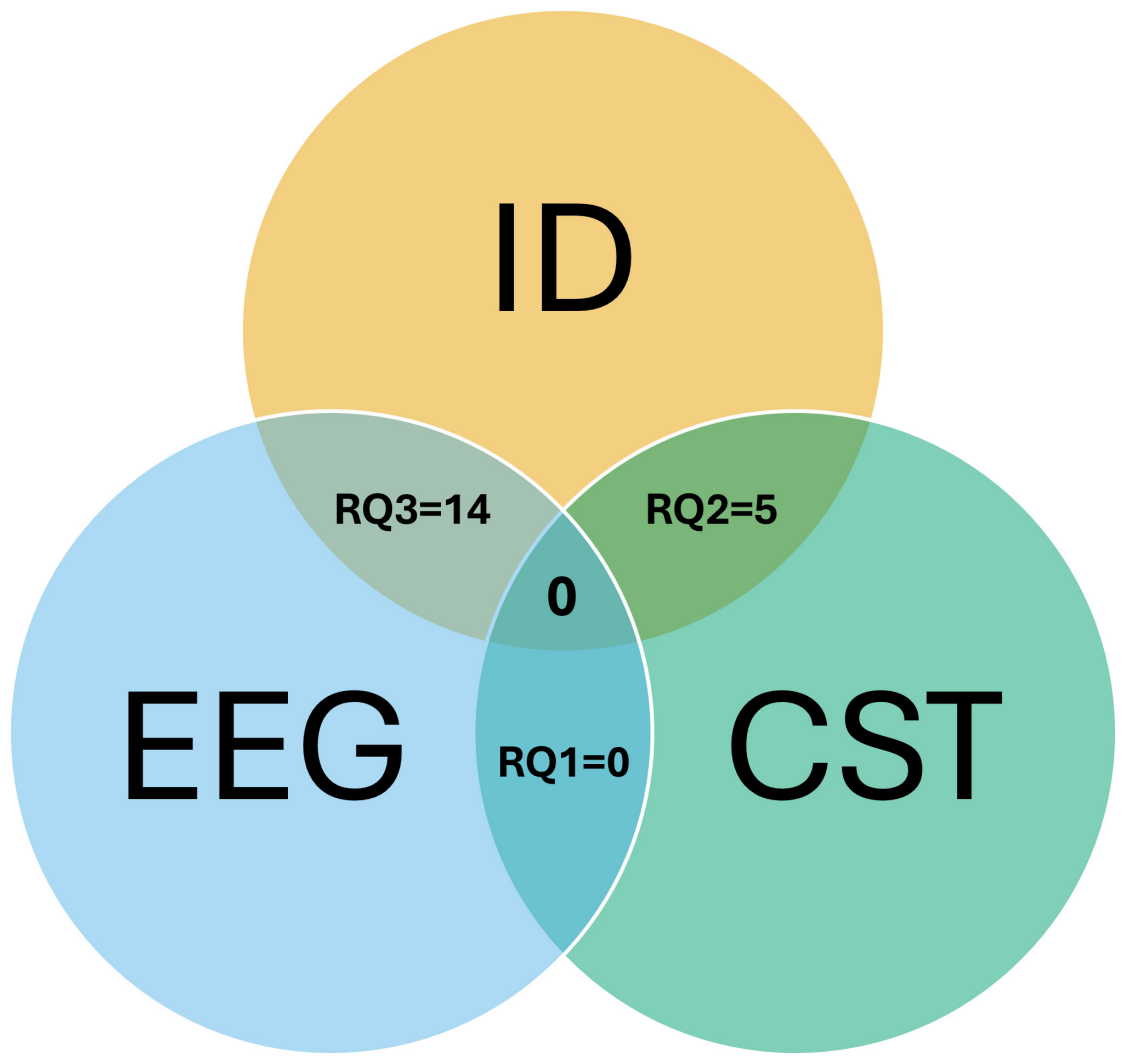
Venn diagram of search concepts and included publications. Overlaps show the number of studies included after screening for each combination: EEG-ID = 14; CST-ID = 5; EEG-CST = 0; EEG-CST-ID = 0.

Articles were searched according to the specific criteria for each RQs. The results retrieved for each stream were: (RQ1) EEG and CST (*n* = 1), (RQ2) CST and ID (*n* = 42), and (RQ3) EEG and ID (*n* = 732). After going through the selection process, final articles were retrieved. RQ1, which focused on usage of EEG in CST research, did not yield any articles that fulfilled the criteria. RQ2, exploring the ID literature and CST, yielded five articles from both peer reviewed sources and gray literature. Finally, RQ3, which explored how EEG has been used to evaluate cognitive and social outcomes in ID, yielded 14 articles which fulfilled the inclusion criteria. The results are arranged according to each RQ. Since only RQ2 and RQ3 yielded results, there are two main sections in this results section. The findings, connections and the gaps will be discussed in the discussion section. Figure 3 below shows the Venn diagram of the intersection for the three search streams.

### 3.1 Overview of CST studies in people with ID

All work in CST involving people with ID took place in the UK and Ireland (Figure 2A). Published studies so far have reported largely on the feasibility of CST for people with mild and moderate ID, without examining outcomes, and with relatively small sample sizes (*N* ranged from 5 to 34), with no large-scale trial yet conducted. Of the five published studies, two studies included people with ID with a diagnosis of dementia (Ali et al., 2022, 2025), one study included people with and without a dementia diagnosis (MacHale et al., 2024) while the other two studies only included people without a dementia diagnosis (Dunne et al., 2025; Hardiman et al., 2025). Full details of the participants are shown in Table 2.

**Table 2:** Participant overview of included studies in CST and ID.

Study	Age criteria	Population	Dementia
Dunne et al. (2025)	DS: 35 years and over	DS and other etiology	No
	Non-DS: 50 years and over		
Hardiman et al. (2025)	20–45 years	DS only	No
Ali et al. (2025)	18 years and over	DS and other etiology	Yes
MacHale et al. (2024)	DS: 35 years and over	DS and other etiology	Mix
	Non-DS: 50 years and over		
Ali et al. (2022)	40 years and over	DS and other etiology	Yes

#### 3.1.1 Feasibility findings

Overall, studies showed that CST is feasible to conduct in adults with ID. Although recruitment was challenging in most studies, retention rates were high, and participant dropout was minimal. Among participants who attended CST within their day services, retention reached 100% (Dunne et al., 2025; Hardiman et al., 2025). Attendance was also exceptionally high across group CST studies, with most participants attending at least ten sessions on an average (Ali et al., 2025; Dunne et al., 2025; Hardiman et al., 2025).

The adaptability of CST has been identified as a key factor enabling engagement and adherence (MacHale et al., 2024). Facilitators are encouraged to ensure that activities are appropriately matched to the group's abilities, as tasks that are too difficult or too easy may reduce enjoyment and participation (Ali et al., 2022; Dunne et al., 2025). Reported reasons for absences included transportation difficulties (Ali et al., 2025) and scheduling conflicts or illness (Dunne et al., 2025).

Only two studies have evaluated the fidelity of intervention delivery, which ensures the intervention was delivered exactly as it was designed, such as following the same structure and using it consistently. Available evidence suggests that fidelity was lower in individual CST compared with group CST, which showed good agreement between observers and facilitators (Ali et al., 2022, 2025).

#### 3.1.2 Outcome measures

Each study determined its sample size based on feasibility objectives rather than formal power calculations, with participant numbers ranging from 5 to 34. Four articles (4/5) provided outcome measures, while the remaining article was a qualitative study and, therefore, was not included in this subsection MacHale et al., (2024). Although outcome measures varied across studies, cognition was the most frequently assessed domain. Table 3 shows more details on the outcome measures evaluated by each study. Table 4 shows that findings related to cognitive outcomes were mixed: some studies reported no significant differences between the intervention and control groups Ali et al., (2022); Dunne et al., (2025), whereas others found positive changes favoring the intervention Ali et al., (2025); Hardiman et al., (2025). A list of the different measurement instruments for cognition can also be seen in Table 4.

**Table 3:** Outcome measures reported by each study.

Studies	Cognitive	Quality of life	Adaptive behavior	Depression	Subjective wellbeing	Health and social care resource use
Dunne et al. (2025)	X	X	X			
Hardiman et al. (2025)	X		X		X	
Ali et al. (2025)	X	X		X		X
Ali et al. (2022)	X	X	X			

An “X” indicates that the outcome was assessed/reported in the study. Outcomes include cognition, quality of life, adaptive behavior, depression, subjective wellbeing, and health/social care resource use.

**Table 4:** Cognitive measures used and significance results for each study.

Study	Cognitive measure used	Significant
Dunne et al. (2025)	CAMCOG-DS	No
Hardiman et al. (2025)	FULD retrieval	Yes
	FULD repeated retrieval	No
	Verbal fluency- FULD verbal fluency	No
Ali et al. (2025)	The Severe Impairment Battery (SIB)	No
The Dementia Questionnaire for people with Learning Disabilities (DLD)	Yes
Ali et al. (2022)	The Cambridge Cognitive Examination for Older Adults with Down Syndrome (CAM-COG-DS)	No
	The modified memory for objects tests from the neuropsychological assessment of dementia in intellectual disabilities battery	No
		The Cognitive Scale for Down Syndrome (CSDS)	No

Quality of life, measured using the Quality of Life in Alzheimer's Disease (QoL-AD) scale, also showed higher scores for participants in the intervention group at follow-up, although these differences did not reach statistical significance (Ali et al., 2022, 2025). Additional improvements were observed in communication, socialization, and adaptive behavior domains (Hardiman et al., 2025).

### 3.2 EEG and ID

#### 3.2.1 EEG measurements in ID

EEG was used to assess cognitive or social outcomes in 14 studies in individuals with ID. All records were individual EEG studies and no hyperscanning setups were identified. Five studies used EEG as a diagnostic/biomarker approach for AD in DS (DSAD) (Musaeus et al., 2019a,b, 2021; Salem et al., 2015; Victorino et al., 2023), of which one was a review (Victorino et al., 2023). The remaining nine studies investigated EEG in ID unrelated to dementia, most commonly in DS (*n* = 6) (Anagnostopoulou et al., 2021; Babiloni et al., 2010; Hamburg et al., 2019, 2021; Katada et al., 2000; Lubińska-Kościółek et al., 2018), followed by Fragile X syndrome (FXS; *n* = 2) (Picciuca et al., 2023; Schmitt et al., 2022) and other/unspecified IDs (*n* = 1) (Palix et al., 2020). Of these nine, one evaluated EEG as a diagnostic tool for ID itself (Lubińska-Kościółek et al., 2018) and the other eight used EEG for basic mechanistic research or intervention assessment.

**Figure 4:**
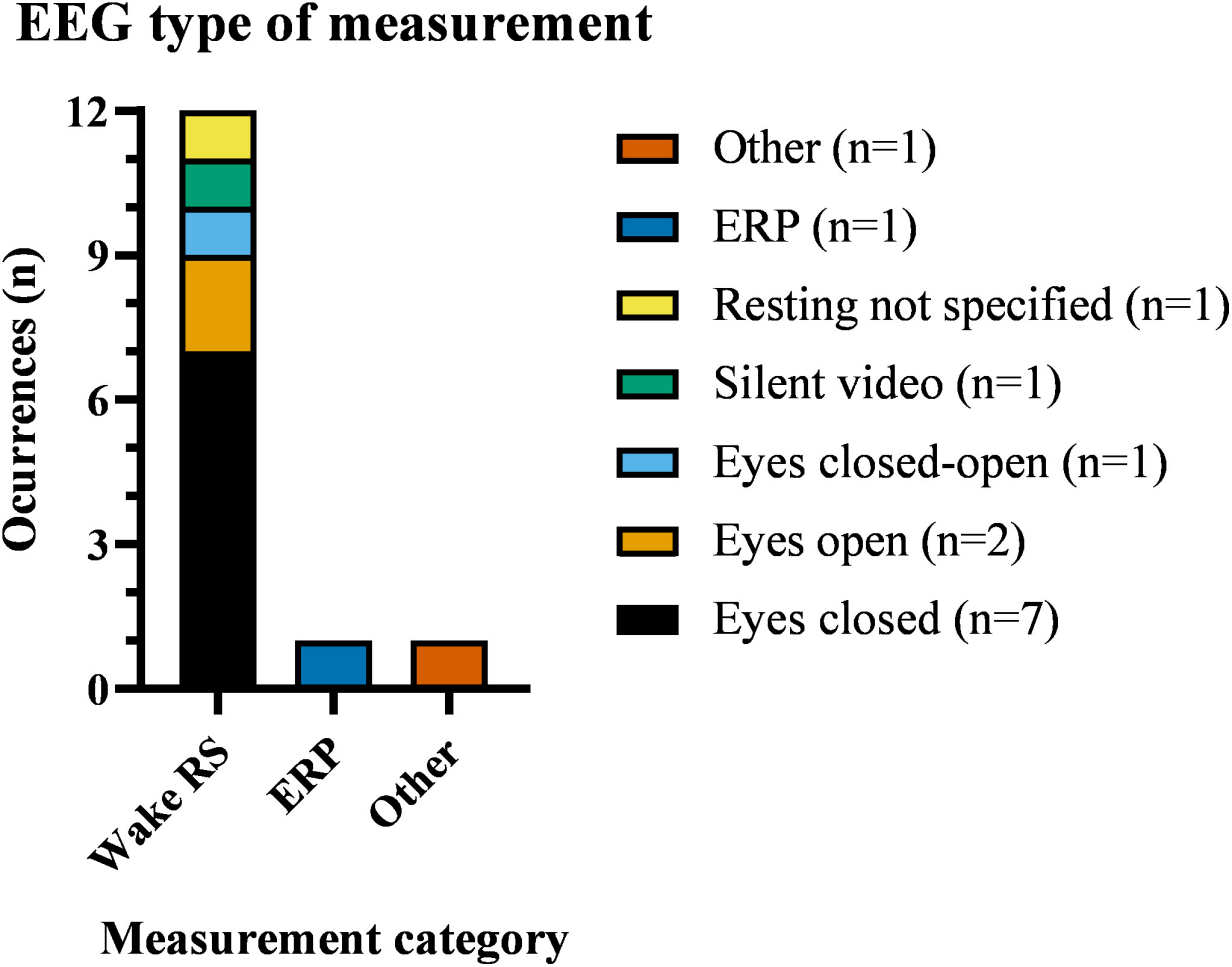
Distribution of EEG measurement types across the included studies. Bars indicate occurrences per category: wake resting-state (stacked by protocol: eyes closed, *n* = 7; eyes open, *n* = 2; alternating eyes closed-open, *n* = 1; silent-video resting, *n* = 1; resting not specified, *n* = 1), ERP (*n* = 1), and Other (*n* = 1). Wake resting-state predominated, with eyes-closed the most common protocol.

Figure 4 shows that experimental tasks were predominantly wake resting state (wake RS; *n* = 12/14), with one ERP (TMS-evoked potential) study (Picciuca et al., 2023) and one review not specifying a task (Victorino et al., 2023). Among the resting-state protocols, eyes-closed (EC) was most common (*n* = 7) (Babiloni et al., 2010; Hamburg et al., 2019, 2021; Katada et al., 2000; Musaeus et al., 2019a,b, 2021), followed by eyes-open (EO; *n* = 2) (Anagnostopoulou et al., 2021; Palix et al., 2020); one study used alternating EC–EO blocks (*n* = 1) (Lubińska-Kościółek et al., 2018), one study used a silent video to maintain engagement during rest (Schmitt et al., 2022), and one did not specify the resting-state modality (Salem et al., 2015).

#### 3.2.2 EEG metrics in ID

Among the five DSAD studies, two primary studies used spectral power (frequency-domain) measures as the main EEG outcome (i.e., power spectral density), derived absolute and/or relative band power and related spectral summaries Musaeus et al., (2019b); Salem et al., (2015). A third paper was a review that primarily discussed and interpreted spectral power findings, as well as related measures such as cross-frequency coupling (CFC), of which phase-amplitude coupling (PAC) was predominantly used, as reported in the literature Victorino et al., (2023). One study focused on microstate analysis Musaeus et al., (2019a), and one centered on undirected functional connectivity, i.e., coherence, total coherence, and weighted phase lag index (wPLI) Musaeus et al., (2021).

Among the nine studies on ID unrelated to dementia, analytic approaches were heterogeneous. The most frequent were power/spectral analyses (*n* = 5) (Babiloni et al., 2010; Hamburg et al., 2019, 2021; Katada et al., 2000; Lubińska-Kościółek et al., 2018), followed by source imaging utilizing low-resolution electromagnetic tomography (LORETA; *n* = 2) (Anagnostopoulou et al., 2021; Babiloni et al., 2010). Regression models appeared four times, including general linear model (*n* = 1) (Hamburg et al., 2019), linear mixed-effects model (*n* = 1) (Schmitt et al., 2022) and multiple linear regression (*n* = 2) (Hamburg et al., 2019; Palix et al., 2020). Connectivity metrics were less common: directed using phase transfer entropy (PTE; *n* = 1) (Anagnostopoulou et al., 2021) and dynamic causal modeling (DCM; *n* = 1) (Hamburg et al., 2019); and undirected with debiased wPLI (dwPLI; *n* = 1) (Schmitt et al., 2022). Graph theory (Anagnostopoulou et al., 2021), entropy-based complexity (ApEn) (Palix et al., 2020), TMS-evoked potentials (TEP) (Picciuca et al., 2023), and correlation analysis (partial Spearman) (Schmitt et al., 2022) were only used in one article (*n* = 1) each. Because several studies reported more than one metric, counts refer to occurrences rather than unique studies. Table 5 summarizes occurrences across all 14 articles; Supplementary Table S4 provides useful definitions of each metric used in the previous studies.

**Table 5:** Summary of the metrics' occurrences across the 14 EEG in ID studies included in the review.

EEG metrics	Occurrences (*n*)	EEG metrics	Occurrences (*n*)
**Connectivity (undirected)**	**4**	**CFC/PAC**	**1**
dwPLI	1	**Microstate analysis**	**1**
wPLI	1	**(TEP)**	**1**
Coherence	1	**Correlation analysis**	**1**
Total coherence	1	Partial Spearman	1
**Connectivity (directed)**	**2**	**Regression models**	**4**
PTE	1	General linear model	1
DCM	1	Linear mixed-effects model	1
**Graph theory**	**1**	Multilinear model	2
**Entropy estimation**	**1**	**Power/spectral analysis**	**8**
**Source analysis (LORETA)**	**3**		

Bold formatting indicated the metric category.

#### 3.2.3 EEG as a biomarker for dementia in DS

Studies that used EEG as a biomarker in participants with DSAD compared to DS without AD dementia found that the centroid frequency of the theta band is decreased in several regions of the brain, predominantly in electrodes Fp1 and F7 (left frontotemporal region), indicating that theta activity is shifted toward lower frequencies within the theta range as revealed by spectral and power analysis (Salem et al., 2015). Complementing this, Victorino et al. (2023), reported increased slow-band activity, especially in the theta range, consistent with a broader pattern of EEG slowing in DSAD. Some studies (Musaeus et al., 2019b; Victorino et al., 2023) also showed decreased global power of alpha waves and/or reduced slow alpha band activity. Alpha coherence was found to be decreased between occipital-temporal electrodes and between central-frontal electrodes, especially in the right hemisphere; theta coherence also showed decreases between central-frontal-occipital electrodes; and delta coherence was overall increased across all regions of the brain. wPLI showed similar patterns, with decreased global alpha and theta, and increased global delta, predominantly between frontal-central-parietal electrodes. However, neither coherence nor wPLI showed any significance across electrodes after correcting for multiple comparisons (Musaeus et al., 2021). A decreased global alpha/delta ratio was also found in participants with DSAD compared to DS (Musaeus et al., 2019b). One study (Victorino et al., 2023) suggested that altered theta-gamma PAC may be one of the earliest EEG signatures of AD and may therefore serve as an adjuvant tool for detecting cognitive decline in DSAD. The only study that analyzed EEG microstates (Musaeus et al., 2019a) did not find any significant differences between DS and DSAD.

#### 3.2.4 Neural mechanisms in ID

In people with ID, compared to typically developing controls (TDC), one study (Babiloni et al., 2010) reported a decrease in the alpha band amplitude of the posterior brain sources. Hamburg et al. (2021) reported not only lower power and peak amplitude and greater peak frequency variance in the alpha band of frontal and occipital regions, but also lower beta power. Peak frequencies were also found to vary with age, with dominant peaks in people with DS in their twenties found at 9 Hz in frontal, occipital and central brain regions, and with a distinct decrease with age (Katada et al., 2000). By contrast, increased delta and theta power of frontal and occipital regions (Hamburg et al., 2021); frontal and parietal regions (Lubińska-Kościółek et al., 2018); and global delta band amplitude and power (Babiloni et al., 2010) has been shown in some studies. This effect was not significantly correlated with intelligence quotient (IQ) (Babiloni et al., 2010).

A connectivity study (Schmitt et al., 2022) reported that, after controlling for non-verbal IQ, increased error rates on executive function tasks were associated with increased gamma-band and reduced alpha-band dwPLI connectivity across all frontal regions and across hemispheres compared to TDC, although dwPLI values were not correlated to IQ. Similarly, connectivity analysis showed a significant reorganization of cortical connections using graph theory, with increased global efficiency (GE) and transitivity (TS), and decreased characteristic path length (CPL); and increased PTE connectivity in several networks including the dorsal attention network, the visual and fronto-parietal network, the default-mode-network and the ventral attention network (Anagnostopoulou et al., 2021). One study (Palix et al., 2020) reported that task reaction times were negatively correlated with IQ; however, ApEn values of parietal electrodes in the ID group did not differ from those in the control group. In a TMS-EEG study (Picciuca et al., 2023), right hemisphere dorsolateral pre-frontal cortex (dlPFC) stimulation resulted in a brief post-treatment reduction of TMS-evoked activity over left frontal sites, which was consistent with better post-treatment language performance.

#### 3.2.5 Association with psychological assessments

Three out of the four studies (excluding the review paper) investigating DSAD explored associations between EEG activity and the Dementia Screening Questionnaire for Individuals with Intellectual Disabilities (DSQIID). No significant correlation was found between alpha coherence and DSQIID scores (Musaeus et al., 2021). Decreased frequency in the theta band was correlated with increased cognitive impairment as measured by DSQIID scores (Salem et al., 2015). The alpha/delta ratio was significantly correlated with DSQIID scores (Musaeus et al., 2019b). Musaeus et al. (2019a) explored microstates in individuals with DSAD, which is a type of analysis that shows topographical maps associated with resting state networks. Associations between DSQIID scores and microstate temporal parameters, focusing on the duration of canonical microstate classes A (linked to phonological processing) and D (linked to attentional processes) (Britz et al., 2010), revealed a small positive correlation between DSQIID scores and occurrence of microstate A, although this was not statistically significant. By contrast, in people with ID without dementia, one study (Hamburg et al., 2019) reported that higher scores on the Kaufmann Brief Intelligence Test, Second Edition (KBIT-2), were associated with higher frontal alpha peak amplitude and higher theta–alpha band power across distributed regions.

## 4 Discussion

The aim of this scoping review was to map how hyperscanning can be used for CST in people with ID. A first preliminary scoping search combining the term Hyperscanning (and related synonyms) with CST OR ID (and related terms) revealed a clear evidence gap in literature with relation to objective measurement: no studies were identified that used hyperscanning as an outcome measure in neither CST nor ID research. A second, broader preliminary scoping search combining EEG (and related synonyms) with both CST AND ID (and related terms) likewise retrieved no studies. In light of this gap, the final search strategy operationalised the research aim by separating it into three complementary query streams aligned with the three research questions, broadening the scope to capture how CST and EEG have been studied to date and to highlight where evidence remains limited. We found that only a small number of studies have implemented CST for people with ID (five studies), and even fewer have used EEG to evaluate CST. Our search identified only one such record, a protocol for a randomized controlled trial which was excluded during the screening stage because it did not fulfill the eligibility criteria (Jornkokgoud et al., 2025). CST therefore sits at the core of the current limitations, indicating that both EEG in CST and CST in ID populations are underexplored areas that require further study to support meaningful conclusions. Consequently, this scoping review aims to guide future research in this developing field.

CST is a psychosocial intervention for people with dementia (Spector et al., 2003) initially developed as a group intervention that was further adapted for individual CST (Orrell et al., 2012). It has been shown to improve both cognition and social outcomes such as depression (Saragih et al., 2022), quality of life, language, and activities of daily living of people with dementia (Chen, 2022). However, its use in ID has only recently emerged. As is typical for an emerging field, the available articles are feasibility-based, which included participants both with and without a diagnosis of dementia. These studies suggest that CST for people with ID is a feasible intervention, with high engagement and adherence (Ali et al., 2025; Dunne et al., 2025; Hardiman et al., 2025). This high level of participation is likely attributable to the strong engagement and enjoyment reported by both participants and facilitators (Dunne et al., 2025; MacHale et al., 2024).

While very limited, results using traditional outcomes measures of cognition, quality of life and adaptive functioning were mixed. Some preliminary evidence suggests CST may lead to improvement in cognition, quality of life, communication, and adaptive behavior; however, studies have not yet been powered to examine this. Cognitive tests can be hindered by both floor and ceiling effects in this population (Aschenbrenner et al., 2021), and while there are validated cognitive measures (Ivain et al., 2025), these were not designed for testing pre-post intervention only a few weeks apart. Bias due to learning effects can thus compromise results. In addition, such cognitive measures often exclude those with more severe or profound level of ID, excluding them from this type of research. This highlights the need for a complementary objective outcome metric that can be correlated with traditional measures. CST trials have observed increased interaction and enthusiasm in group environments (Coen et al., 2011), which is also reflected in ID literature, noting CST as a medium to socially connect with others (MacHale et al., 2024). Since the group dynamics and enjoyment aspects are extensively cited in CST literature both in general population and in ID, there is scope for measuring this social interaction objectively.

EEG has long been considered a valuable method for investigating brain function due to its excellent temporal resolution. Several studies have leveraged this to examine brain-wave alterations in people with ID, aiming to characterize these patterns, identify biomarkers of cognitive impairment, and clarify underlying mechanisms. However, none of these studies assessed the feasibility of using EEG in a population with ID in terms of acceptability, who are at increased risk of dementia, and thus who may benefit the most. Some reported difficulties in complying with tasks such as resting state, owing to participants' inability to remain still, relaxed, or with eyes closed (or open) for extended periods (Anagnostopoulou et al., 2021; Babiloni et al., 2010; Hamburg et al., 2019, 2021; Katada et al., 2000; Musaeus et al., 2019a,b, 2021; Salem et al., 2015; Schmitt et al., 2022). Consequently, some participants' data were lost due to excessive noise, artifacts, drowsiness, and related issues (Musaeus et al., 2019a,b, 2021; Salem et al., 2015). Furthermore, none of the presented studies reported retention rates, although the great majority were not interventional or longitudinal. Regarding EEG metrics, most studies relied on spectral analysis as the primary approach, suggesting a shift in power and amplitude from the alpha band toward the theta and delta bands. The correlation between IQ and these changes remains uncertain, with some studies showing significant differences between control and ID groups (Hamburg et al., 2019), while others do not (Babiloni et al., 2010). Nonetheless, these studies provide valuable insight into how EEG can be used to derive metrics related to participants' cognitive abilities at an individual level.

In collective settings, hyperscanning is a neuroimaging method first coined in 2002 that has gained prominence over the past decade (Montague et al., 2002). Notably, the number of publications has increased markedly in the last 5 years, showcasing how hyperscanning can be used to study neural underpinnings of the brain during social interaction. Several studies have examined social interaction across a range of social situations (e.g., child-parent interactions, familiar vs. unfamiliar conditions) and task conditions (e.g., passive tasks, interactive and non-interactive economic games) (Kinreich et al., 2017; Léné et al., 2021; Wang et al., 2018). Most of these studies focus on inter-brain synchrony (IBS) based on connectivity metrics that reflect the state of social facilitation (Bakhshayesh et al., 2019; Czeszumski et al., 2020). Some studies have even begun comparing modern interaction methods such as gaming or online texting with traditional in-person interaction (Wikström et al., 2022; Schwartz et al., 2024). Recent studies have used hyperscanning to investigate brain synchrony/connectivity in vulnerable populations, especially those on the autism spectrum (Kang et al., 2023; Key et al., 2022; Chen et al., 2025; Chuang et al., 2023; Moreau et al., 2024; Ranjabaran et al., 2025). One study even used hyperscanning to explore the brain dynamics between a person with dementia and a music therapist (Maidhof et al., 2023). Nonetheless, the application of this promising method to people with ID has yet to be reported.

Studies included in this review indicate that EEG is a promising method for studying brain activity in people with ID, and that CST can be implemented in this population. Hyperscanning can investigate brain dynamics across individuals and across intervention time points, which needs to be explored as a complementary approach for socio-psychotherapy, group-based interventions such as CST. However, some practical issues should be addressed to avoid pitfalls and fill current gaps in EEG research in ID. Feasibility metrics are essential to evaluate tolerability and effectiveness, for example, questionnaires on participants' opinions about EEG and the comfort of the device. These may be complemented with retention metrics such as absences per session and drop-out rates, which help characterize participant behavior and inform protocols to mitigate non-attendance and attrition. Moreover, certain tasks may be difficult to measure due to participant behavior, potentially limiting EEG use during CST sessions. Tasks should therefore be carefully selected or adapted, with consideration of alternatives to traditional resting-state EEG. Examples include shorter, repeated blocks (Hamburg et al., 2019); longer blocks to select high-quality segments (Katada et al., 2000; Musaeus et al., 2019a,b, 2021); and an appropriate choice among eyes-open, eyes-closed, or other resting-state variants such as silent video (Schmitt et al., 2022) to keep participants engaged. In addition, tasks adapted from CST protocols must be designed with the heightened risk of erratic movements in mind, as this population may be prone to sudden movements or touching electrodes that can contaminate EEG data. To mitigate these and other artifact-inducing effects, such as speech production, preprocessing pipelines should be rigorously implemented, and protocol tasks should be adapted to EEG constraints, minimizing electrical interference and high-movement activities, and avoiding any activities that could damage EEG equipment.

Adapted CST protocols for people with ID have been proposed, and both the use of EEG and CST has been increasing for this population in the last decade (Figure 2B). These protocols typically include 10 min of introduction, 25 min of games, and 10 min of summary and closing. Given the heterogeneity of tasks across sessions, data collection at specific time points is limited to certain session components. Accordingly, EEG analyses for social facilitation metrics could focus on repeated elements across sessions to establish periods of comparison at different timepoints of an intervention (e.g., an interactive segment during the introduction such as a group song). Brain recordings during the game segment may also yield essential information on how engagement progresses within a session and could provide intra-brain metrics across sessions. On this note, EEG may provide valuable information on the session tasks, allowing to optimize CST protocols for people with ID. As shown in Figure 4, resting states have extensively been used as the main measurement in EEG in ID. While not an appropriate measure to probe active social interaction, resting states are fundamental for comparing task recordings with a baseline where minimal interaction or engagement is expected. Future studies may consider the benefit of adding a resting state EEG at the beginning of each session and incorporating another at the end to assess data quality across the session and to compare non-interactive baseline engagement possibly inflated during a session. In addition, some studies have found that resting states such as group mindfulness improve social behavior and connectedness (Deng et al., 2023, 2024; Matiz et al., 2021).

CST interventions, developed by Spector et al. (2000) and based on reality orientation, have proven to benefit quality of life, social relationships and communication (Gibbor et al., 2021). The CST protocol is designed to be flexible, allowing facilitators to adapt the tasks to the group requirements, as long as they do not deviate from the core skeleton of the protocol sustained in the 18 key principles and the recommended structure in terms of length of course and sessions. These key principles are in-line with social cognitive theories (SCT) based on the claim that it shows a direct correlation between a person's perceived self-efficacy and behavioral change (Bandura, 2001). CST tasks may benefit participants targeting essential behaviors present in SCT, such as social connectedness, enjoyment, building and strengthening relationships, and continuous mental stimulation. The behaviors promoted by CST are closely tied to social cognitive elements such as social perception, social understanding, social decision-making (Arioli et al., 2018), presenting EEG hyperscanning as an eligible method to investigate the social dynamics of CST participants.

Some studies have used IBS to study naturalistic spoken communication in situations of knowledge sharing, turn-taking, problem-solving tasks, and teamwork (Kelsen et al., 2022; Réveillé et al., 2024), key group interactions involved in CST. To quantify the degree of social facilitation in these contexts, phase-based IBS metrics that reduce spurious synchrony, such as PLI/wPLI (and the debiased wPLI), have been used in the past (Ahn et al., 2018). In CST, these metrics could be prioritized for repeated interactional elements across sessions (e.g., a standardized group song during the introduction), enabling within-subject comparisons across intervention timepoints, while treating the heterogeneous “games” block primarily as a window into within-session dynamics (e.g., how engagement and coupling evolve over time). Importantly, dyadic synchrony measures such as dwPLI remain pairwise; they can still be extended to groups by computing all pairwise inter-brain edges and summarizing them as “hyperbrain” networks using graph-theory descriptors (Toppi et al., 2016). Given that CST is often group-based, genuinely multivariate approaches are also valuable. These include multivariate coherence or more exploratory extensions that include information-theoretic dependence measures (e.g., mutual information and conditional variants) to probe shared vs. unique information across multiple brains.

However, the analysis of highly interactive CST components requires a baseline where near-zero engagement and synchrony is expected. Achieving a “true” resting state may be challenging in this population; therefore, complementary within-person indices of arousal/involvement may be useful for quality control and interpretation. Such metrics may be based on spectral analysis across participants' brains (e.g., assessing engagement via spectral power ratios in relevant regions of interest such as the mentalizing system and mirror neuron system) (Wang et al., 2018). For example, EEG ratio indices such as beta/alpha or beta/(alpha+theta) have been used as markers of attentional engagement/mental effort (Marcantoni et al., 2023), and could help contextualize low-quality baseline segments (e.g., elevated arousal during “rest”) vs. more interactive periods (e.g., group song or game play). These ratio indices could also enable knowing whether higher individual involvement is associated with stronger IBS during interaction. In a group-based CST session, higher engagement might correlate with higher IBS, and a drop in engagement from one participant may be reflected in the pairwise (or even multivariate) IBS metrics of the task. Nonetheless, engagement may not necessarily translate to social facilitation, therefore engagement indexes should be considered as complementary metrics to support IBS metrics. To interpret those correlations meaningfully, IBS and involvement measures should be anchored to established behavioral and subjective indicators of CST participation. Thus, relating derived EEG metrics to traditional outcomes (such as questionnaires assessing connectedness, engagement, enjoyment, and other aspects of the session), is essential, as this enables characterization of the session context and supports validation of hyperscanning methods in CST for people with ID.

## 5 Limitations

This scoping review has several limitations that should be acknowledged. First, none of the EEG studies employed a group design or a hyperscanning setup. All EEG measurements were conducted on a one-to-one basis. As a result, any future hyperscanning work in this population will be exploratory. Second, the evidence identified across each stream was limited. This scarcity of studies represents an important finding, highlighting a significant gap in the literature underscoring the need for further investigation. However, the considerable heterogeneity in study design among the limited available studies hampers the ability to make clear recommendations for future methodological choices particularly regarding measurement types and analytic approaches. This challenge is compounded by the small sample sizes reported in many studies which were often statistically underpowered. Such limitations restrict the ability to detect meaningful effects and reduce the generalizability of findings. Finally, most studies included participants with DS, resulting in an underrepresentation of individuals with ID of other etiologies. This could be primarily due to the well-established genetic risk for AD. However, different types of ID share characteristics such as developmental and cognitive delay, difficulty in carrying out activities of daily living and overlapping structural brain features (e.g., cerebellar hypoplasia, characterized by reduced cerebellar volume). Findings from this scoping review identified similarities in EEG profiles between individuals with Fragile X syndrome and DS, suggesting the potential for generalizability across ID etiologies. However, the extent to which these patterns vary across different levels of ID remains unclear and represents an important direction for future research. From the context of CST, the efficacy of the intervention is not likely dependent upon the etiology of ID; however, if different etiologies exhibit distinct neural pathways through CST, EEG may be well-positioned to make this distinction with the help of stratified analysis. This analysis could also show how hyperscanning performs in ID of different severity levels. Thus, future research should prioritize more diverse samples to determine how individual EEG and hyperscanning methods may work for different causes and severity levels of ID.

## 6 Conclusion

This scoping review shows that CST has been implemented in a small number of feasibility studies in people with ID, and that EEG has been used in limited samples of people with ID and DSAD. However, there are currently no completed trials using EEG to evaluate CST in ID and no hyperscanning studies in this population. These findings highlight a substantial knowledge gap. Individual EEG has been used to evaluate cognitive outcomes in people with ID, yet limited work has been conducted to derive behavioral metrics. Hyperscanning has been used in studies of social interaction and represents a potential avenue for future research seeking to examine cognitive and social processes alongside traditional CST outcome measures. However, hyperscanning feasibility, tolerability and validity in people with ID and dementia must first be established.
