# Supplementary material for: EEG hyperscanning in intellectual disability: a scoping review with implications for cognitive stimulation therapy
Source: Front Neuroergon. 2026 Apr 13;7:1757738. doi: 10.3389/fnrgo.2026.1757738 (PMC13111357; doi:10.3389/fnrgo.2026.1757738)
Supplement: Supplementary file 1 [file Table_1.docx]

**Search Strategy**

**Date- 15-08-2025**

**Main concepts**

|  | *Concept* | *Terms* | | | | |
| --- | --- | --- | --- | --- | --- | --- |
| #1 | **Hyperscanning** | Hyperscanning | Group EEG | Collective EEG |  |  |
| #2 | **EEG** | EEG | Electroencephalography | Electroencephalogram |  |  |
| #3 | **Cognitive stimulation therapy** | Cognitive stimulation therapy | CST |  |  |  |
| #4 | **People with intellectual disability** | Intellectual disability | Learning disability | Down syndrome | Intellectual development disorder | Intellectual Impairment |

**Source Index Terms in Databases**

|  | *Concept* | *Index Term: CINAHL* | *Index Term: Medline* | *Index Term: PsycINFO* | *Index Term: EMBASE* |
| --- | --- | --- | --- | --- | --- |
| #1 | **Hyperscanning** | No index term available | No index term available | No index term available | 'hyperscanning'/exp |
| #2 | **EEG** | (MH "Electroencephalography") | (MH "Electroencephalography") | DE "Electroencephalography" | 'electroencephalogram'/exp |
| #3 | **Cognitive stimulation therapy** | No index term available | No index term available | DE "Cognitive Stimulation Therapy" | 'cognitive stimulation therapy'/exp |
| #4 | **People with intellectual disability** | (MH “Intellectual disability” / MH “person with intellectual disability”/ MM Down Syndrome) | (MH "Intellectual Disability") OR (MH "Learning Disabilities") OR (MH "Persons with Intellectual Disabilities")  OR (MH “Down Syndrome”) | DE "Intellectual Development Disorder" | 'intellectual impairment'/exp |

**Super Search**

|  | *Concept* | Combining keywords and index terms |
| --- | --- | --- |
| #1 | **Hyperscanning** | TI ("Hyperscanning" OR "Hyperbrain" OR "Interbrain connectivity" OR "Interbrain synchrony" OR "Interbrain coherence" OR "Brain-to-brain") OR AB ("Hyperscanning" OR "Hyperbrain" OR "Interbrain connectivity" OR "Interbrain synchrony" OR "Interbrain coherence" OR "Brain-to-brain") |
| #2 | **EEG** | TI ("Electroencephalography" OR “EEG” OR "Electroencephalogram") OR AB ("Electroencephalography" OR “EEG” OR "Electroencephalogram") |
| #3 | **Cognitive stimulation therapy** | TI ("Cognitive stimulation therapy" OR "CST") OR AB ("Cognitive stimulation therapy" OR "CST") |
| #4 | **People with intellectual disability** | TI ("Intellectual disability" OR "person with intellectual disability" OR "learning disability" OR "Down syndrome" OR "Intellectual development disorder" OR "Intellectual impairment") OR AB ("Intellectual disability" OR "person with intellectual disability" OR "learning disability" OR "Down syndrome" OR "Intellectual development disorder" OR "Intellectual impairment") |
