# Supplementary material for: EEG hyperscanning in intellectual disability: a scoping review with implications for cognitive stimulation therapy
Source: Front Neuroergon. 2026 Apr 13;7:1757738. doi: 10.3389/fnrgo.2026.1757738 (PMC13111357; doi:10.3389/fnrgo.2026.1757738)
Supplement: Supplementary file 2 [file Data_Sheet_1.docx]

Supplementary table 1. Data extraction (Cognitive stimulation therapy in Intellectual disability)

| **Title** | **Country** | **Aim of study** | **Population** | **Outcome measures** | **Feasibility findings** | **Outcome measure results** |
| --- | --- | --- | --- | --- | --- | --- |
| Hardiman, S., Cousins, R., Ryan, A., Kennedy, M., Hagan, L., & Santos, F. H. (2025). Proactive Cognitive Stimulation for Younger Adults With Down Syndrome. A Feasibility Randomised Control Trial. *J Appl Res Intellect Disabil*, *38*(5), e70120. https://doi.org/10.1111/jar.70120 | Ireland | To measure feasibility and acceptability in terms of recruitment and retention rate, barriers to participation, barriers to attending the CST programme, session satisfaction and engagement, CST programme fidelity, and the resource-cost of running this adapted CST programme within one community-based intellectual disability service. | Adults with Down syndrome without dementia. | FULD recall trial  The Vineland Adaptive Behaviour Scales 3rd edition Domain-Level Parent/Caregiver Form  Personal Wellbeing Score (PWS) | There was high retention rate (100%), strong attendance across 86% of the sessions, high satisfaction reported by participants (95%). Facilitators rating for participant engagement showed similar result. There was high interest, communication, enjoyment, and mood. A notable concern was low recruitment rate (16%). | Between-group findings showed no difference at baseline but the intervention group scored significantly higher on communication and overall adaptive behaviour composite scores than the control group. At 4-month follow-up the intervention group had higher communication, socialisation, and overall adaptive scores compared to the control group.  Within-group comparison showed that the scores of FULD retrieval increased significantly from baseline to post-intervention for the CST group. But this increase was not observed in the control group. |
| Dunne, P., McCallion, P., Synnott, I.F., Pavithra, P., Lavelle, L., O’Loughlin, C., Galligan, L., O’Reilly, L., Hynes, M. & McCarron, M. Engaging the Brain Through Cognitive Stimulation Therapy (CST). A Feasibility Randomised Controlled Trial for Adults with an Intellectual Disability. Avista, August 2025. | Ireland | To investigate the feasibility of implementing group Cognitive Stimulation Therapy (CST) with adults who have an intellectual disability and  are at risk of developing dementia. | Aged 35+ (Down syndrome), or  aged 50+ (intellectual disability) without dementia | The Cambridge Cognitive Examination for Older Adults with Down Syndrome (CAM-COG-DS)  Health-Related Quality of Life for Individuals with Intellectual and Developmental Disability  Older Americans Resources and Services (OARS) Activities of Daily Living scale | This study reported CST to be a feasible and acceptable intervention to be run in a day service. It found 100% adherence, and 0% drop-out. It also found high engagement and enjoyment amongst participants. | Cognitive scores improved over time for both group but there were no differences between the groups. Other measures did not report any significant changes. |
| Ali, A., Francis, C., Hoare, S., Carter, J., Goulden, N., Clarke, C. S., Charlesworth, G., Hoare, Z., Acton, D., Khanum, S., Onafalujo, A., Jejeloye, A., Brackley, K., Aguirre, E., & Spector, A. (2025). Group cognitive stimulation therapy for people with intellectual disability and dementia: feasibility randomised controlled trial. *BJPsych Open*, *11*(5), e168. https://doi.org/10.1192/bjo.2025.10764 | United Kingdom | To assess the feasibility of participant recruitment and retention, the appropriateness of outcome measures, and the feasibility of group CST (adherence, fidelity, acceptability), as well as the feasibility of collecting data for an economic evaluation. | Participants with intellectual disability diagnosed with dementia. | The Severe Impairment Battery  The Dementia Questionnaire for People with Learning Disabilities  The Quality of Life in Dementia proxy questionnaire  EuroQol-5 Dimensions-3 Levels (EQ-5D-3L) version  EQ-5D-5L proxy version 1  The Carer Supplement to the Glasgow Depression Scale for people with a Learning Disability | Of 61 eligible participants, 46 consented and 41 completed baseline assessments; 34 were randomised, with withdrawals mainly due to illness, hospitalisation, or logistical issues. Adherence was moderate, with 16 participants attending 64% of available sessions (nine attended ≥10 sessions). Fidelity was high (mean score = 25.4; 75.9%), with good facilitator–observer agreement (κ = 0.79). Qualitative findings indicated social interaction, improved communication, memory, and sleep as key benefits. Barriers included travel distance, carer availability, and fatigue from double sessions, while enablers were facilitator adaptability and carer involvement. | The DLD and the QOL-AD proxy showed positive changes in favour of the intervention group, whereas the results of the SIB and GDS-Proxy were in favour of the control arm. The  intraclass correlation values for the GDS-LD proxy and QoL-AD proxy were negligible (<0.001), although those for the DLD (0.098)  and SIB (0.29) were larger. |
| MacHale, R., NíNeill, E., Wyer, C., Corley, E., & McGuire, B. E. (2024). Preliminary feasibility study of a cognitive stimulation therapy programme for older adults with an intellectual disability. *Journal of Applied Research in Intellectual Disabilities*, *37*(5), e13291. https://doi.org/https://doi.org/10.1111/jar.13291 | Ireland | to explore the feasibility of CST for older adults with intellectual disability to support active ageing | Aged 35+ (Down syndrome), or  aged 50+ (intellectual disability) with or without dementia | Qualitative | Overall, data analysis suggests the CST group was acceptable with no drop-out and high level of attendance. Facilitators also rated high participant interest, communication, enjoyment and mood throughout the group. | The qualitative analysis generated three themes: brain health, connecting with others, and barriers and enablers |
| Ali, A., Brown, E., Tsang, W., Spector, A., Aguirre, E., Hoare, S., & Hassiotis, A. (2022). Individual cognitive stimulation therapy (iCST) for people with intellectual disability and dementia: a feasibility randomised controlled trial. *Aging & Mental Health*, *26*(4), 698-708. https://doi.org/10.1080/13607863.2020.1869180 | United Kingdom | To examine the feasibility, acceptability and fidelity of individual Cognitive  Stimulation Therapy (iCST) in people with intellectual disability (ID) and dementia | This study aimed to recruit 40 dyads (person with intellectual disability and their carer) with 20 dyads in each study arm. This sample size was decided for pragmatic reasons. | The Cambridge Cognitive Examination for Older Adults with Down Syndrome (CAM-COG-DS)  Modified Memory for Objects Test  The Cognitive Scale for Down Syndrome (CSDS)  Alzheimer's Disease Cooperative Study - Activities of Daily Living (ADCS-ADL)  Quality of Life-Alzheimer’s Disease Scale | Recruitment and retention rates were high, with 83% of eligible dyads enrolling, 87.5% completing midpoint, and 97.5% completing endpoint assessments. Adherence averaged 70%, with 10% of dyads completing all activities, 60% completing more than half, and 30% completing less than half. Most participants omitted the warm-up (87.5%) and orientation (91.7%) components, while 41.1% used reminiscence activities. Acceptability was high, with carers and participants reporting satisfaction with study procedures, session duration, and overall engagement, though some activities were described as challenging. | There were no significant differences between the iCST and the control group in the outcome measures for cognition and adaptive functioning at 11-weeks or 21-weeks, after adjusting for baseline scores. There were no differences in quality of life at 11-weeks, but at 21 weeks, it was higher in the iCST arm compared to the control arm. |
