# Supplementary material for: EEG hyperscanning in intellectual disability: a scoping review with implications for cognitive stimulation therapy
Source: Front Neuroergon. 2026 Apr 13;7:1757738. doi: 10.3389/fnrgo.2026.1757738 (PMC13111357; doi:10.3389/fnrgo.2026.1757738)
Supplement: Supplementary file 3 [file Data_Sheet_2.docx]

Supplementary table 2: EEG in Intellectual disability

| **Title** | **Country** | **Aim of the study** | **Study design** | **Group 1 characteristics** | **Group 2 characteristics** | **Electrode type** | **EEG type** | **Electrode Array** | **Measurements** | **Metrics** | **Assessments** | **Key findings** |
| --- | --- | --- | --- | --- | --- | --- | --- | --- | --- | --- | --- | --- |
| Victorino, D. B., Faber, J., Pinheiro, D., Scorza, F. A., Almeida, A. C. G., Costa, A. C. S., & Scorza, C. A. (2023). Toward the Identification of Neurophysiological Biomarkers for Alzheimer's Disease in Down Syndrome: A Potential Role for Cross-Frequency Phase-Amplitude Coupling Analysis. Aging Dis, 14(2), 428-449. https://doi.org/10.14336/ad.2022.0906 | Brazil | To accumulate evidence for altered theta-gamma phase to amplitude coupling (PAC) as a biomarker for AD, serving as an adjuvant tool for detecting cognitive decline in DS-AD. | Review article of preclinical and clinical evidence. | n/a | n/a | Not mentioned | Not mentioned | n/a | Resting state and simple cognitive / memory / WM tasks. | Classic measures: (1) Absolute / relative band power, (2) mean alpha frequency / alpha slowing, (3) topography / sources of rhythms, (4) synchronization / coupling / coherence of resting rhythms (inter-hemispheric functional coupling and global synchronization correlating), and (5) dynamic causal modeling (DCM).  PAC / CFC-specific metrics: (1) Modulation Index (MI), (2) phase-locking value (PLV)-based PAC, (3) mean Vector Length (MVL) modulation index, (4) phase-amplitude comodulogram, and (5) cross-frequency coupling patterns over time. | n/a | Resting-state EEG in individuals with Down syndrome (DS) shows increased slow-wave activity (theta/delta) and reduced reactive alpha compared to neurotypical adults, mirroring patterns in sporadic Alzheimer’s disease (AD). Adolescents and young adults with DS demonstrate reduced interhemispheric and fronto–posterior coupling of alpha and beta rhythms. Evidence from sporadic AD and mild cognitive impairment highlights disrupted cross-frequency coupling, particularly theta–gamma phase–amplitude coupling (PAC). These findings suggest that EEG slowing and altered PAC in DS may reflect cognitive decline and serve as potential biomarkers for dementia onset, though evidence remains limited. |
| Picciuca, C., Assogna, M., Esposito, R., D'Acunto, A., Ferraresi, M., Picazio, S., Borghi, I., Martino Cinnera, A., Bonnì, S., Chiurazzi, P., & Koch, G. (2023). Transcranial direct current stimulation combined with speech therapy in Fragile X syndrome patients: a pilot study. *Front Neurol*, *14*, 1268165. <https://doi.org/10.3389/fneur.2023.1268165> | Italy | Evaluation of the safety, tolerability, and efficacy of anodal prefrontal transcranial direct current stimulation (tDCS) combined with standard speech therapy to enhance language function in FXS patients. | Pre-post intervention study 5-week design with 45 mintDCS and speech therapy. | FXS Mean age (SD): 26.9 ± 10.11  15 male/1 female  Sample size: 16 | n/a | Wet | Mobile Scalp | The EEG was recorded from 64 sites positioned according to the 10 to 20 International System, using TMS-compatible Ag/AgCl pellet electrodes mounted on an elastic cap. EEG signals were digitized at a sampling rate of 5 kHz. | TMS to EEG acquisition (T0 & T1): Eighty TMS single-pulses at a random ISI of 2-4 s (±20%) over right and left DLPFC (coil over F3 and F4) at 110% of resting motor threshold. EEG recorded from 64 sites (10 to 20) using TMS-compatible Ag/AgCl pellet electrodes and a TMS-compatible DC amplifier (BrainAmp), digitized at 5 kHz. | TMS-evoked potentials (TEPs). | Word, Non-Word, and Phrase Reading Test; Word, Non-Word, and Phrase Writing Test; Word, Non-Word, and Phrase Repetition Test; Word Copy Test; Test for Reception of Grammar – Version 2 (TROG-2) | TMS-EEG analysis showed a significant difference in TMS-evoked potential (TEP) amplitude over the left frontal cortex after treatment compared to baseline. A significant positive cluster relating to the differences before and after treatment was revealed when stimulating the right dorsolateral prefrontal cortex (DLPFC). This cluster involved signal changes in left frontal electrodes (e.g., F3, Fz, C5, AFz) in the time interval of 26 to 51 ms. The researchers interpreted the post-treatment change within this cluster (which was negative on average) as indicating that TEP amplitude in left frontal sites decreased after the treatment, suggesting a decrease in frontal excitability. Time-frequency analysis (oscillatory domain) did not yield statistically significant cluster results for either stimulation condition. Correlational analysis established a relationship between clinical improvements and changes in neurophysiological excitability. Correlational results showed that patients who demonstrated better linguistic performances after treatment had reduced frontal excitability. |
| Schmitt, L. M., Li, J., Liu, R., Horn, P. S., Sweeney, J. A., Erickson, C. A., & Pedapati, E. V. (2022). Altered frontal connectivity as a mechanism for executive function deficits in fragile X syndrome. Mol Autism, 13(1), 47. https://doi.org/10.1186/s13229-022-00527-0 | United States | To examine phase connectivity within the frontal cortex and its relation to performance-based EF measures in order to provide novel insight into the functional relevance of altered alpha and gamma band oscillatory activity in FXS. | case-control design | FXS Mean age (SD): 21 (10.2)  33 male/28 female  Sample size: 61 | Control Mean age (SD) 22.2 (10.7)  41 male/30 female  Sample size: 71 | Not mentioned | Not mentioned | 128-channel EGI HydroCel Geodesic Sensor Net with a sampling rate at 1000 Hz. | Five minutes of continuous resting EEG at a sampling rate at 1000 Hz. | Neural connectivity was assessed using the debiased weighted phase lag index (dWPLI).  Executive function (EF) was measured using eight KiTAP variables.  Partial Spearman was used to correlate frontal phase connectivity and EF performance.  Generalized linear models were used to identify signficant predictors of KiTAP outcomes. | Abbreviated Stanford Binet-5th edition (SB-5) to estimate general cognitive functioning.  Participants completed four subtests: Alertness (processing speed), Distractibility (attention), Go/NoGo (response inhibition), and Flexibility (cognitive flexibility). | Individuals with FXS demonstrated increased gamma band and reduced alpha band connectivity across all frontal regions and across hemispheres compared to TDC. After controlling for nonverbal IQ, increased error rates on EF tasks were associated with increased gamma band and reduced alpha band connectivity. |
| Musaeus, C. S., Salem, L. C., Kjaer, T. W., & Waldemar, G. (2021). Electroencephalographic functional connectivity is altered in persons with Down syndrome and Alzheimer's disease. *J Intellect Disabil Res*, *65*(3), 236-245. https://doi.org/10.1111/jir.12803 | Denmark | To investigate whether EEG functional connectivity could be used as a diagnostic marker for AD in persons with DS. | comparison study | DS (Mean, SD): 47.50 (8.70) | DS-AD (Mean, DS): 51.69 (4.98) | Wet | Not mentioned | Standard 44-channel headbox was used. Each subject was fitted with a cap using silver-silver-chloride-coated electrodes. | Wake resting (30 mins) state from 19 electrodes positioned according to the International 10-20 system. Data were sampled at 1.0 kHz. | Connectivity calculations- Coherence was calculated between each pair of electrodes for each of the following frequency bands: delta (1-3.99 Hz), theta (4-7.99 Hz), alpha (8-12.99 Hz) and beta (13-29.99 Hz).  Total coherence was calculated. The weighted phase lag index was calculated for the same frequency bands as described for coherence. | DSQIID | Decreased alpha and increased delta-  coherence and weighted phase lag index were observed in DS-AD as compared with DS. The decrease in alpha coherence was more marked in the fronto-parietal connections as compared with the temporo-parietal connections. No significant correlation was found between DSQIID and total alpha coherence (P value = 0.095, rho = 0.335). he decreased alpha coherence and weighted phase lag index have previously been found in AD. The increased delta coherence and weighted phase lag index may indicate a different initial neurophysiological presentation as compared with patients with AD or may be a sign of more advanced disease  Coherence results showed that there were significant increases in the alpha band between the occipital and temporal electrodes and the central and frontal electrodes which were most prominent on the right side. There was also a decreased coherence in the theta band between the frontal central, and occipital electrodes and an overall increase in delta coherence. Only a few significant changes and no overall changes were found in the beta band. When correcting for multiple comparisons, no significant electrode-to-electrode comparisons were found. In terms of total coherence, a significant lower total alpha coherence in DS-AD was found compared to the DS group and there were no significant differences in the total fronto-parietal or temporo-parietal coherence. However, there was a larger difference in total alpha coherence in the fronto-parietal as compared with the temporal-parietal coherence  Weighted phase lag index results- found that DS-AD group showed decreased global alpha and increased global delta. The electrode-to-electrode comparisons found that were were more pronounced changes in the frontal, central, and parietal electrodes in the delta band. When correcting for multiple comparisons, no significant electrode-to-electrode comparisons were found.  Correlation with DSQIID- There was no significant correlation between alpha coherence and DSQIID. |
| Hamburg, S., Bush, D., Strydom, A., & Startin, C. M. (2021). Comparison of resting-state EEG between adults with Down syndrome and typically developing controls. *J Neurodev Disord*, *13*(1), 48. https://doi.org/10.1186/s11689-021-09392-z | United Kingdom | To compare EEG activity between adults with DS and TD controls using commonly used frequency band classifications, and both absolute and relative power, in addition to including two scalp regions (occipital and frontal). | Cross-sectional, matched case control observational study. | DS age:16 yeas old and over  13 females/12 males | Control age: 6 - 44 years old  11 Females/ 12 males | Wet | Wired Scalp | 128-channel EEG Geodesic Hydrocel nets (Electrical Geodesics, Inc., Eugene, OR, USA). | In both datasets, electrode impedances were maintained below 50 k during recording.  During the resting-state task, participants of both groups repeated multiple eyes closed (EC) recording blocks. However, the first 11 participants with DS had one continuous 5.5 min EC block, which was then changed to multiple shorter blocks due to poor compliance and drowsiness. | For each individual, absolute and relative power measures for each frequency band of interest were obtained (delta 0.5-4 Hz; theta 4-8 Hz; alpha 8-13Hz; beta 13-30 Hz) for each region (frontal and occipital). Additionally, alpha peak features were calculated. | CAMDEX-DS | Study reported an overall slower EEG spectrum, characterised by higher delta and theta power, and lower alpha and beta power, for both regions in people with DS. Alpha activity in particular showed strong group differences, including lower power, lower peak amplitude and greater peak frequency variance in people with DS. |
| Anagnostopoulou, A., Styliadis, C., Kartsidis, P., Romanopoulou, E., Zilidou, V., Karali, C., Karagianni, M., Klados, M., Paraskevopoulos, E., & Bamidis, P. D. (2021). Computerized physical and cognitive training improves the functional architecture of the brain in adults with Down syndrome: A network science EEG study. *Netw Neurosci*, *5*(1), 274-294. <https://doi.org/10.1162/netn_a_00177> | Greece | To use resting-state EEG network analysis to characterize how a combined physical and cognitive training program affects functional brain network organization in adults with Down syndrome. | single-group, pre- and post-intervention design | DS Age Mean (SD): 29 (11)  6 males/6 females  n=12 | n/a | Not mentioned | Not mentioned | Nihon-Kohden high-density EEG (lab) 128 active scalp electrodes. | Pre- and post-intervention resting-state EEG (eyes open) was recorded for 5 min at 1,000 Hz; electrode impedances were kept lower than 10 kΩ. | Functional Connectivity Metrics- Phase Transfer Entropy (PTE); Resting-State Network (RSN) Connectivity; Node Degree Centrality (DC)  Graph-Theoretical (Network Science) Measures- (1) Global Network Measures: Global Efficiency (GE), Transitivity (TS), Characteristic Path Length (CPL); (2) Local Network Measures: Clustering Coefficient (CC), Betweenness Centrality (BC); (3) Small-Worldness and Hierarchical Organization: Small-World (SW) Measure, Community Detection/Hierarchical Organization | WISC-III: Digits Span, WISC-III: Mazes test, WISC-III: Picture Arrangement, WISC-III: Block Design, Raven's Progressive Matrices, Arm curl test, Timed Up and Go test, Short Physical Performance Battery, 10-meter walk, Back scratch, Sit and reach, Four square step, Stork balance, Body mass index | Psychometric & Physical Outcomes:  Participants showed significant improvements in arm curl performance and faster Timed Up and Go. Cognitive gains were observed in Digit Span Forward, Mazes, and Raven’s AB and total scores; other measures showed no significant changes.  EEG / Network Connectivity (PTE):  Post-intervention EEG revealed reorganization of resting-state networks, with increased connectivity within the dorsal attention network and between VIS, FPN, DMN, VAN, and SMN.  Graph-Theory Metrics:  Global efficiency and transitivity increased, while characteristic path length decreased. Locally, clustering coefficient showed widespread increases across nodes, and betweenness centrality increased in a small subset. Small-worldness rose but remained below the ? > 1 threshold. Community analysis indicated a shift to a more complex hierarchical network structure post-intervention. |
| Palix, J., Giuliani, F., Sierro, G., Brandner, C., & Favrod, J. (2020). Temporal regularity of cerebral activity at rest correlates with slowness of reaction times in intellectual disability. *Clinical Neurophysiology*, *131*(8), 1859-1865. https://doi.org/https://doi.org/10.1016/j.clinph.2020.04.174 | Switzerland | To examine whether behavioral slowness in ID could originate from abnormal complexity in brain signals. | Participants (N = 29) performed a reaction times (RTs) task. Continuous electroencephalogram recording during the resting period was used to quantify brain-signal complexity by approximate entropy (ApEn). | Moderate ID Age Mean (SD): 33 (11)  11 male/3 female  Sample size: 14 | Healthy controls Age Mean (SD): 35 (9)  11 male/4 female  Sample size: 15 | Wet | Wired Scalp | Sixty-four active pin-type electrodes (BioSemi ActiveTwo EEG System acquisition, BioSemi B.V. WG Plein 129 1054SC Amsterdam) had been attached to the Quick-Cap according to the 10 to 20 international position system. | The feedback loop provided by BioSemi ActiveTwo was used to ensure valid measurements. This system allows to avoid any abnormal range before recordings (common mode voltage) and to evaluate the good conductivity of signal at all electrodes to sites (electrodes offset visualization). A continuous electroencephalogram was then recorded during the resting state (awake with eyes open) to be used offline in the estimation of the complexity of EEG resting activity. The EEG data were continuously acquired at a sampling rate of 4096 Hz with ActiView software (version 5.34) through 0.5 to 100 Hz filters. | Entropy estimation (ApEn) was used to calculate complexity of the signals and complemented with spectral and autocorrelation analyses.  Multilinear regression analysis was conducted to predict RTs duration, with IQ, ApEn and age as independent variables. | WAIS-III instrument (Wechsler, 1981)  The RTs task was the Poffenberger paradigm (Poffenberger, 1912). | Reaction Times: When combining all participants (N = 28), RTs were negatively correlated with IQ (r = -0.43, p < .03), indicating that the processing speed tended to decrease with the decline in IQ. The RTs StDevs correlated with IQ even more clearly (r = -0.73; p < .001).  Brain signal complexity: The ApEn values in the ID group did not differ from those in the HC group. When all the participants data were combined, IQ and ApEn did not seem to be related, and no correlation was found between RTs and ApEn. For ID patients, the lower the brain signal complexity, the longer the RTs were. The other predictors were not significant. For the HC group, the model was not significant. |
| Musaeus, C. S., Salem, L. C., Kjaer, T. W., & Waldemar, G. (2019). Microstate Changes Associated With Alzheimer's Disease in Persons With Down Syndrome. *Front Neurosci*, *13*, 1251. https://doi.org/10.3389/fnins.2019.01251 | Denmark | To assess whether microstates could be used to differentiate between persons with DS and persons with DS-AD. | exploratory study design | DS group age [mean (SD) = 47.1 (9.49)  Sample size: 16  EEG data available 10 persons with DS | DS-AD group age[mean (SD) = 51.80 (5.13)  Sample size: 21 | Wet | Wired Scalp | 44-channel headbox. Cap using silver-silver-chloride-coated electrodes and the data sampled at 1 kHz. 19 electrodes positioned according to International 10-20 system. | Wake-resting state. Impendence was aimed to keep below the 10kOhm for all electrodes during the recordings. | The primary set of metrics extracted during the microstate analysis focused on the temporal characteristics of the four archetypical microstates (labeled A, B, C, and D). These were calculated after the global maps were "back-fitted" to each EEG file. | DSQIID | EEG microstate analysis showed no significant difference in overall GEV between DS and DS-AD. DS-AD participants had shorter microstate D and longer microstate A durations, with the largest difference in microstate A. Microstate D1 (left-lateralized) showed greater reduction than D2. Correlations: DSQIID scores were negatively associated with microstate D and positively with microstate A. |
| Musaeus, C. S., Salem, L. C., Sabers, A., Kjaer, T. W., & Waldemar, G. (2019). Associations between electroencephalography power and Alzheimer's disease in persons with Down syndrome. *J Intellect Disabil Res*, *63*(9), 1151-1157. <https://doi.org/10.1111/jir.12627> | Denmark | To examine whether it was possible to identify AD-associated changes (increased high-frequency power and decreased low-frequency  power) in persons with DS-AD compared with DS. | comparison study | DS Age (Mean, SD): 48.4 (8.88)  DS (Female/Male): 6/9  Recruited-  DS: n= 16  Included in analysis-  DS: n= 15 | DS-AD Age (Mean, SD): 52.1 (5.44)  DS-AD (Female/Male): 3/17  Recruited-  DS-AD: n= 21  Included in analysis-  DS-AD: n= 20 | Wet | Not mentioned | Standard 44-channel headbox was used. Each subject was fitted with a cap using silver-silver-chloride-coated electrodes. | 30-minutes wake resting-state was measured from 19 electrodes positioned according to international 10-20 systems. | Power was calculated in each of the following frequency bands: delta (1-3.99 Hz); theta (4-7.99 Hz); alpha (8-12.99 Hz); and beta (13-29.99 Hz).  Global power was calculated, and the alpha/delta ratio was calculated by dividing the global alpha band with the global delta band. | DSQIID | The absolute power analysis after correcting for multiple comparisons found that no electrode comparisons were significantly different between DS and DS-AD.  There was a significant decrease in the global alpha power for the DS-AD group, compared to the DS group. This was also found for the alpha/delta ratio.  The alpha/delta ratio was significantly correlated to the DSQIID.  No significant differences were found in eyes open condition but there was a tendency for decreased alpha and increased theta power for the DS-AD group compared to the DS group. |
| Hamburg, S., Rosch, R., Startin, C. M., Friston, K. J., & Strydom, A. (2019). Dynamic Causal Modeling of the Relationship between Cognition and Theta-alpha Oscillations in Adults with Down Syndrome. *Cereb Cortex*, *29*(5), 2279-2290. https://doi.org/10.1093/cercor/bhz043 | United Kingdom | To offer insights at the level of canonical microcircuits (CMCs) into the neuronal architectures of adults who present with both ID and a genetic susceptibility for dementia. | Cross-sectional, observational correlational, single-group. | Down syndrome age Mean (SD): 30.92 (11.03)  17 male/19 female  n=36 |  | Wet | Wired Scalp | Appropriately sized EGI hydrocel high density sensor nets (containing 128 channel silver-silver chloride electrodes). | Electrode impedances were maintained below 50 k  The initial eyes-closed resting-state EEG paradigm consisted of continuous recording for 5.5 min (i.e., whole-block recording) and partitioning the 5.5-min recording into 30 s blocks with a short break (of variable length according to each participant) between blocks (i.e., split-block recording). | A scalp-wide SPM of power in the combined theta-alpha (4-13 Hz) range was generated, and regression was used to identify significant associations between raw KBIT-2 score and theta-alpha power across the scalp. This analysis was conducted using a general linear model.  Model comparison metric: They specified candidate families where between-subject effects could modulate forward, backward, or intrinsic self-inhibitory connections (alone or in combination) and used Bayesian Model Reduction and (negative) free-energy to pick the best-evidence model. | Cambridge Examination of Mental Disorders of Older People with Down Syndrome and Others with Intellectual Disabilities (CAMDEX-D).  The Kaufmanns Brief Intelligence Test Second Edition (KBIT-2) | Higher KBIT-2 was associated with higher frontal alpha peak amplitude and higher theta to alpha band power across distributed regions. Modelling this association with DCM revealed intrinsic self-inhibition was the key network parameter underlying observed differences in 4-13 Hz power in relation to KBIT-2 and age. In particular, intrinsic self-inhibition in right V1 was negatively correlated with KBIT-2. |
| Lubińska-Kościółek, E., Zielińska, J., & Wołoszczuk, K. (2018). Psychological diagnosis and quantitative electroencephalography analysis in cognitive rehabilitation for people with down syndrome. *Hrvatska Revija Za Rehabilitacijska Istraživanja*, *54*(2), 39-48. https://doi.org/10.31299/hrri.54.2.4 | Poland | (1) Determining bioelectrical function patterns in the brain of a male respondent with Down syndrome; (2) making a quantitative and qualitative diagnosis of a studied person with intellectual abilities; and (3) using the results from these studies to highlight indications for carrying out neurotherapy and educational rehabilitation. | Descriptive observational study (single case).  The first two measurements took place over two consecutive days, and the third one after six days. | Down syndrome  n=18  All males | n/a | Wet | Wired Scalp | 19-electrode system, arranged according to the international system of 10 - 20. | Impedance kept lower than 5 kOhm.  Recording was done for four minutes in the resting state with opened eyes and with closed eyes. | QEEG allows us to quantify the electrical activity of the brain. | Snijders-Oomen Non-verbal Intelligenztest (SON-R), | The recording of the EEG signal with closed eyes showed an increase in the activity in the alpha range and its decline when the eyes were opened. The distribution of the power spectrum of the EEG signal from both measurements with open eyes shows that the highest values were visible in the delta and theta waves in the central line at electrode Fz and Pz and in electrode P3. The study recorded increased signal strength in delta and theta bands in the area of the central and medial frontals, as well as in the parietal area of the respondent brain. |
| Salem, L. C., Sabers, A., Kjaer, T. W., Musaeus, C., Nielsen, M. N., Nielsen, A. G., & Waldemar, G. (2015). Quantitative Electroencephalography as a Diagnostic Tool for Alzheimer's Dementia in Adults with Down Syndrome. *Dement Geriatr Cogn Dis Extra*, *5*(3), 404-413. https://doi.org/10.1159/000438857 | Denmark | To examine the value of qEEG in the diagnostic evaluation of dementia in patients with Down syndrome (DS). | This is an explorative case-control study with two groups: DS and DS-AD. | DS Age: (Mean, range): 48.5 (38-68).  DS (Male to female ratio)- 16:10  Sample size  Recruited-  DS: n=16 | DS-AD (Mean, range): 52.5 (40-63)  DS-AD(Male to female ratio)- 18:3  Sample size  Recruited- DS-AD: n=21 | Wet | Not mentioned | The study used a standard 44-channel head box with each participant fitted with a cap using silver-silver-chloride-coated electrodes. 9 electrodes positioned according to the International 10-20 system (i.e. Fp1, Fp2, F3, Fz, F4, F7, F8, T3, T4, C3, Cz, C4,T5, T6, P3, Pz, P4, O1, O2). . Data were sampled at 1.0 kHz and bandpass filtered at 0.5-70.0 Hz. | 30-mins EEG recording was taken in wake resting-state. | Four qEEG parameters were derived from the PSD: centroid frequency, peak frequency, absolute power, and relative power. | DSQIID | Centroid frequency in the theta-1 band was significantly reduced in the left frontotemporal region (Fp1–F7) in individuals with DS-AD compared to those with DS. No significant group differences were observed for peak frequency, absolute, or relative power across other frequency bands. Regression analysis showed that theta-1 centroid frequency at F7–T3 accounted for 32% of the variance in DSQIID scores, with correlations above 0.52 between reduced theta-1 frequency and greater dementia severity, particularly in the left frontotemporal region. |
| Babiloni, C., Albertini, G., Onorati, P., Muratori, C., Buffo, P., Condoluci, C., Sarà, M., Pistoia, F., Vecchio, F., & Rossini, P. M. (2010). Cortical sources of EEG rhythms are abnormal in down syndrome. *Clin Neurophysiol*, *121*(8), 1205-1212. https://doi.org/10.1016/j.clinph.2010.02.155 | Italy | To investigate whether the dominant resting-state alpha rhythms are greater in amplitude in subjects with down syndrome compared to control participants. | Observational case-control | DS Age- Mean, SEM: 22.8 years ± 0.7  25 male/20 female  n=45 | Age-matched normal control Mean, SEM: 22.4 years ± 0.5.  25 male/20 female  n=45 | Not mentioned | Not mentioned | 19-channel following the international 10/20 system. | EEG resting state at 256 Hz sampling rate for 5 minutes with eyes-closed. Electrooculography was also collected to monitor eye movements. | LORETA was used for the estimation of cortical sources of EEG rhythms. The cortical LORETA solutions predicting scalp EEG spectral power density were regularized to estimate distributed rather than punctual EEG source patterns. | WISC-R; Wechsler, 1982); The Full-scale, Verbal and Performance Intelligent Quotient (FIQ; VIQ; PIQ) | LORETA analysis showed that individuals with Down syndrome (DS) had reduced alpha1, alpha2, and beta1 source power, especially in central, parietal, occipital, temporal, and limbic regions, compared with controls. DS also showed increased occipital delta activity. These differences were significant across multiple regions (p < 0.01–0.000001) and were not related to age or IQ, indicating a consistent pattern of reduced posterior alpha/beta sources and elevated delta activity in DS. |
| Katada, A., Hasegawa, S., Ohira, D., Kumagai, T., Harashima, T., Ozaki, H., & Suzuki, H. (2000). On chronological changes in the basic EEG rhythm in persons with Down syndrome - with special reference to slowing of alpha waves. *Brain Dev*, *22*(4), 224-229. https://doi.org/10.1016/s0387-7604(00)00107-8 | Japan | The authors tried to know specificity of aging in persons with Down syndrome (DS) from the aspect of electroencephalograph (EEG) frequency changes through the cross-sectional and longitudinal studies, in comparison with normal persons as well as those with mentally retardation except the Down syndrome (non-DS MR). | cross-sectional and longitudinal studies, in comparison with normal persons as well as those with mental retardation except the Down syndrome (non-DS MR). | Down syndrome age range: 8 - 55 years old (n=265)  Intellectual disability non-DS age range: 7 - 58 years old (n=242) | Healthy persons: 2 - 59 years old (n=239) | Not mentioned | Not mentioned | EEGs were led from six midline-sagittally arranged locations equally spaced on the scalp. Along the total length from the nasion to the inion, the other points were spaced every 15% intervals, that is, the fronto-central, central, and centro-parietal locations. Bilaterally linked ear lobes were used as the reference. | EEGs were recorded from the subject sitting down on an armchair with eyes closed. Recording was made for 5 to 10 min to obtain the least 3 min record in a waking state without conspicuous artifacts on visual inspection. | Spectrum analysis: the dominant frequency was decided from the peak of the mean spectrum. This study focused mainly on the records from the frontal, central and occipital locations. | n/a | In individuals with Down syndrome (DS), EEG showed age-related slowing of the dominant rhythm. Cross-sectionally, an 8 Hz peak emerged earliest in the central/frontal regions (30–34 yrs) and peaked in the occipital region at 40–44 yrs, indicating progressive slowing beginning in early adulthood. Longitudinally, all participants in their 20s had 9 Hz rhythms, but frequencies <9 Hz became common in the 30s (50%), predominant in the 40s (79%), and remained frequent in the 50s. In contrast, adults with non-DS intellectual disability showed no clear 8 Hz dominance across age groups, indicating less pronounced slowing. |
