# Supplementary material for: EEG hyperscanning in intellectual disability: a scoping review with implications for cognitive stimulation therapy
Source: Front Neuroergon. 2026 Apr 13;7:1757738. doi: 10.3389/fnrgo.2026.1757738 (PMC13111357; doi:10.3389/fnrgo.2026.1757738)
Supplement: Supplementary file 4 [file Data_Sheet_3.docx]

Supplementary table 3. EEG metrics used in intellectual disability studies.

| **EEG Metric** | **Definition** | **Reference** |
| --- | --- | --- |
| Weighted Phase Lag Index (wPLI) | An extension of PLI that quantifies non-zero-lag phase synchronization while weighting each phase lead/lag observation by the magnitude of the imaginary part of the cross-spectrum, so phase differences with larger non-zero imaginary components contribute more strongly than near-zero/weak contributions. | Vinck et al., 2011 |
| Debiased Weighted Phase Lag Index (dwPLI) | A bias-reduced estimator of wPLI intended to mitigate finite-sample inflation. It is obtained by computing the imaginary cross-spectral samples, taking their average (signed) imaginary component, and normalizing by the average magnitude of those imaginary components (often expressed in a squared/debiased form), yielding a weighted non-zero-lag synchrony measure with reduced small-sample bias. | Vinck et al., 2011 |
| Coherence | A frequency-domain measure of coupling that quantifies how consistently two EEG signals maintain a stable phase and amplitude relationship within a band (via the normalized cross-spectrum). | Bowyer, 2016 |
| Total Coherence | A band-specific global summary of functional coupling, computed as the mean of pairwise coherence values across a predefined set of electrodes (or electrode pairs), yielding an overall “how coherent is the network” index for that frequency band. | Musaeus et al., 2021 |
| Phase Transfer Entropy (PTE) | An information-theoretic directed connectivity metric that estimates frequency-specific information flow by computing transfer entropy on phase time-series extracted from band-limited neuronal signals, yielding the strength and direction of coupling between signal phases and remaining relatively robust to realistic noise and linear mixing. | Lobier et al., 2014 |
| Dynamic Causal Modelling (DCM) | A Bayesian, biophysically grounded generative modelling framework that estimates directed (effective) connectivity between neuronal sources by inverting a spatiotemporal state-space model linking source dynamics to EEG/MEG sensor data via a forward (lead-field) model, enabling inference and comparison of competing network architectures. | Kiebel et al., 2008 |
| Graph Theory | An approach that represents EEG-derived connectivity as a graph—a set of nodes (electrodes or brain regions) and edges (functional/effective connections)—implemented as an adjacency matrix and summarized with metrics that describe the brain’s global and local network organization. | Vecchio et al., 2017 |
| Entropy Estimation | A family of measures (typically Shannon/information-entropy–based) that quantify the uncertainty/irregularity of an EEG signal (or a derived representation such as the PSD), with higher entropy for more random/unpredictable activity (e.g., noise-like) and lower entropy for more regular/predictable patterns (e.g., near-sinusoidal). | Sleigh et al., 2005 |
| Source Analysis (LORETA) | An EEG inverse-modelling method that estimates a 3D distribution of cortical current density from multichannel scalp potentials by imposing a smoothness constraint (minimizing the second spatial derivative so neighbouring voxels are similar), yielding low-resolution tomographic maps of grey-matter activity (often assessed per frequency band). | Dattola et al., 2020 |
| Cross-Frequency Coupling (CFC) | A class of metrics capturing interactions between EEG frequency bands; Phase-Amplitude Coupling (PAC) specifically quantifies the statistical dependence whereby the phase of a low-frequency rhythm modulates the amplitude/power of a higher-frequency component, supporting integration across temporal and spatial scales. | Canolty and Knight, 2010 |
| Microstate Analysis | A method that segments multichannel EEG into successive, brief periods (~60–120 ms) of semi-stable scalp potential topographies (“microstates”) and quantifies their occurrence and temporal dynamics, interpreted as discrete building blocks of large-scale, coordinated brain network activity. | Michel and Koenig, 2018 |
| TMS-Evoked Potentials (TEP) | EEG voltage deflections time-locked to a TMS pulse that index the brain’s immediate, sequential response to cortical stimulation—reflecting local activation and its propagation through networks—though they can be contaminated by peripheral sensory-evoked activity from the stimulation. | Sulcova et al., 2022 |
| Partial Spearman | A rank-based (nonparametric) partial correlation that quantifies the monotonic association between two EEG-derived variables (e.g., band power/connectivity/behavioral score) while statistically controlling for one or more covariates (e.g., age, head motion, vigilance). | Schober et al., 2018 |
| General Linear Model | A linear regression framework applied voxel-/sensor-/time–frequency–wise to EEG features, fitting a linear model at each point and using SPM-style statistical inference (e.g., correction for multiple comparisons). |  |
| Multilinear Model | Models a continuous EEG-derived outcome (e.g., spectral features, entropy, RT-linked EEG measures) as a linear combination of multiple predictors/covariates, estimating each predictor’s unique contribution. |  |
| Linear Mixed-Effects Model | A linear regression that includes fixed effects (predictors of interest) and random effects (e.g., subject-specific intercepts/slopes) to account for repeated measures or hierarchical EEG data. |  |
| Power / Spectral Analysis | Methods that estimate how EEG signal power is distributed across frequencies (power spectral density) and summarize it as band power or spectral peaks using Fourier- or wavelet-based approaches. |  |
