# Supplementary figures and images for: EEG hyperscanning in intellectual disability: a scoping review with implications for cognitive stimulation therapy
Source: Front Neuroergon. 2026 Apr 13;7:1757738. doi: 10.3389/fnrgo.2026.1757738 (PMC13111357; doi:10.3389/fnrgo.2026.1757738)

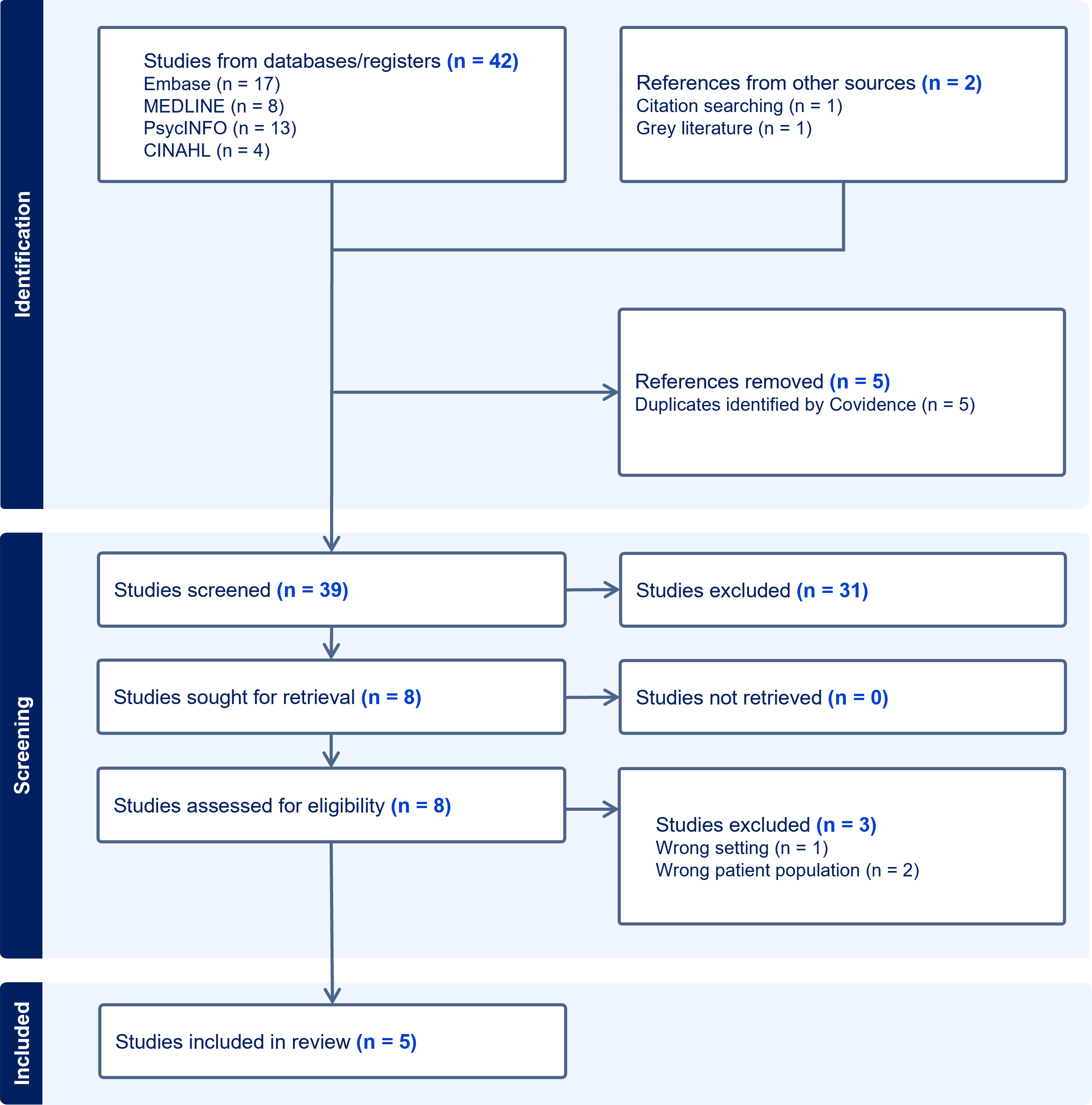

Supplement: Supplementary file 5 [file Image_1.png]

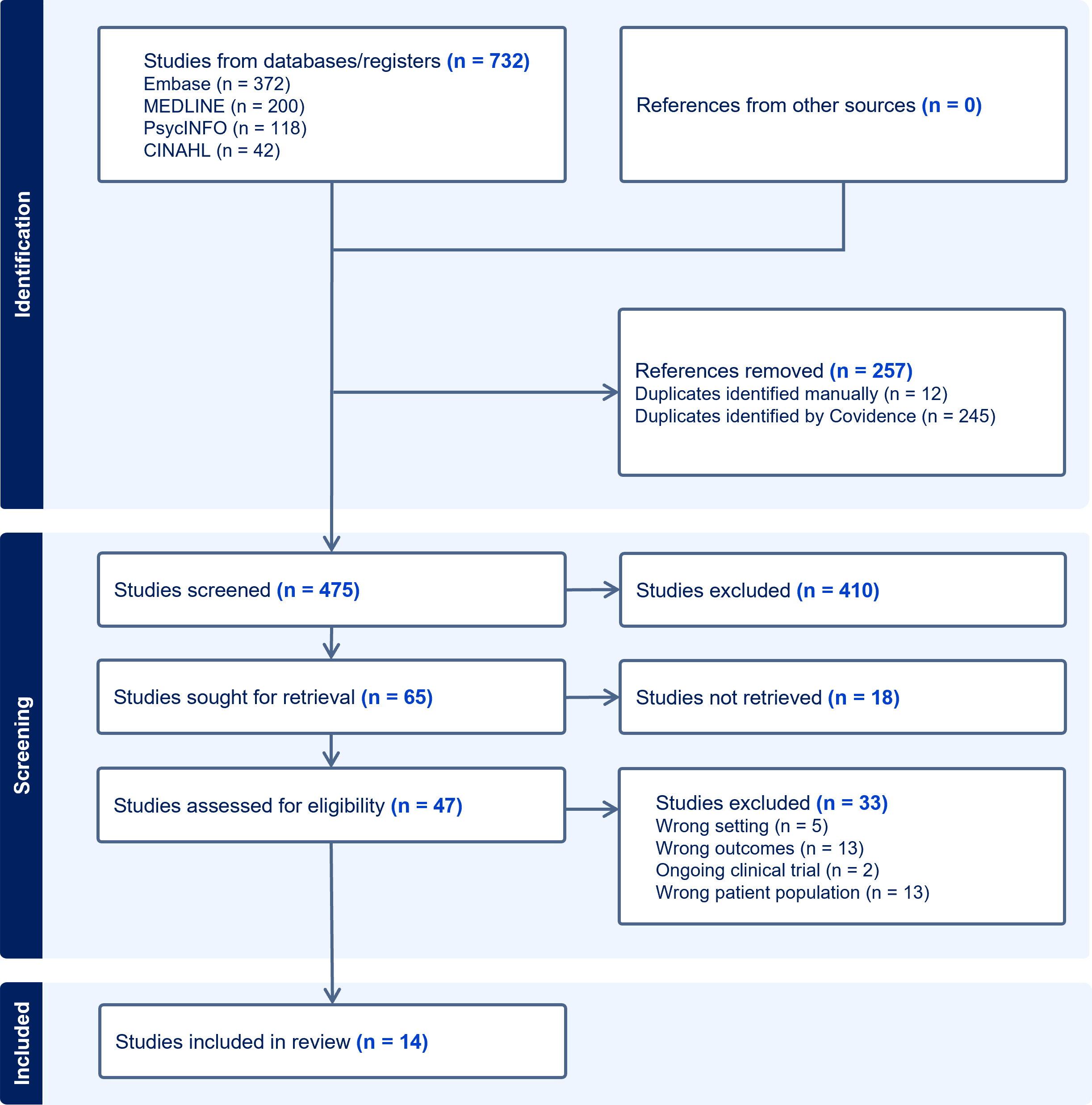

Supplement: Supplementary file 6 [file Image_2.png]

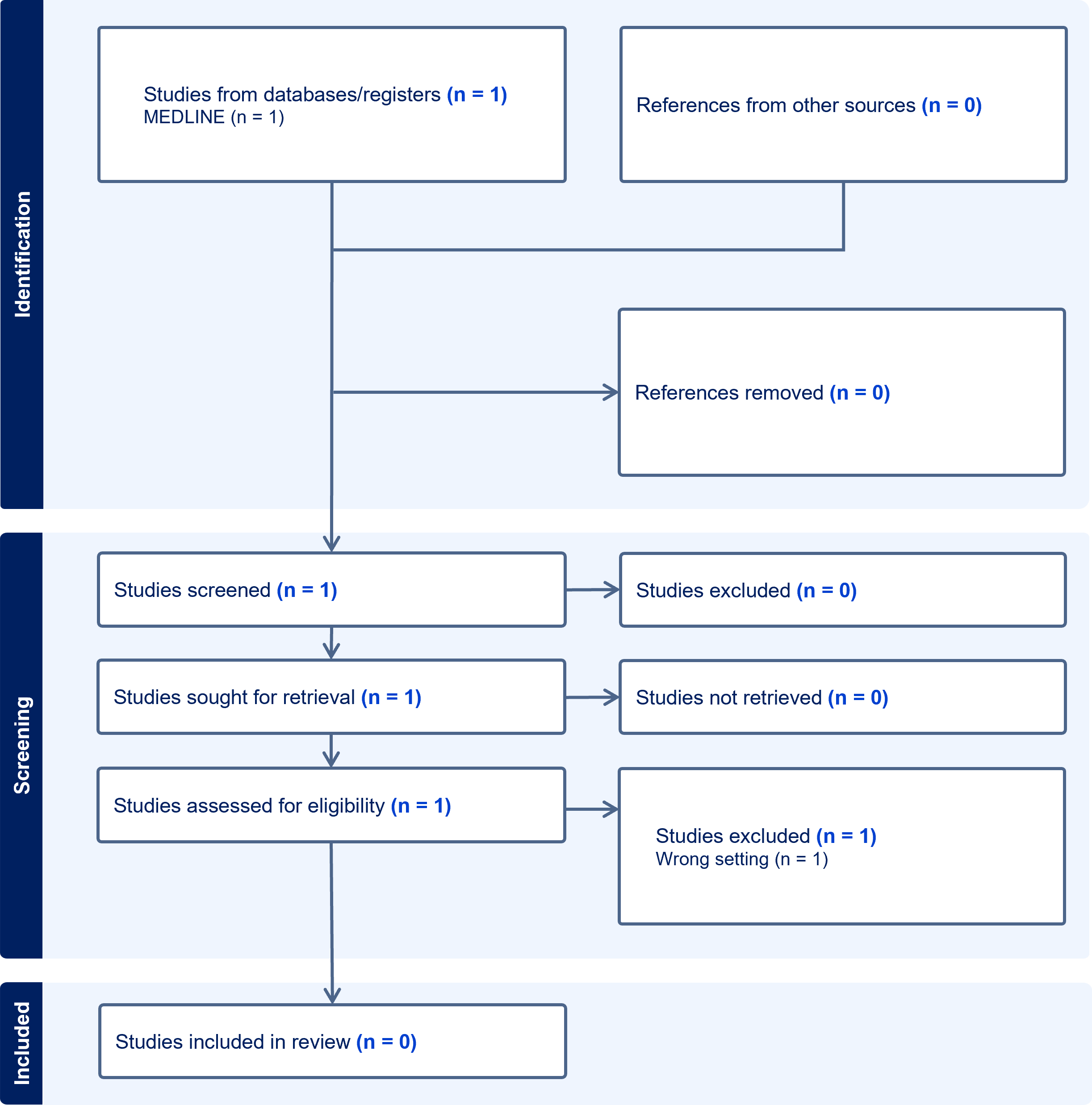

Supplement: Supplementary file 7 [file Image_3.png]
